# Immunogenicity of autologous and allogeneic human primary cholangiocyte organoid cellular therapies

**DOI:** 10.1016/j.xcrm.2025.102205

**Published:** 2025-07-07

**Authors:** Sandra Petrus-Reurer, Winnie Lei, Olivia Tysoe, Maelle Mairesse, Adrian Baez-Ortega, Julia Jones, Thomas Tan, Sylvia Rehakova, Krishnaa T. Mahbubani, Cara Brodie, Namshik Han, Inigo Martincorena, Catherine Betts, Ludovic Vallier, Kourosh Saeb-Parsy

**Affiliations:** 1 Department of Surgery, University of Cambridge and NIHR Cambridge Biomedical Research Centre, Cambridge CB2 0QQ, UK; 2 Milner Therapeutics Institute, University of Cambridge, Cambridge CB2 0AW, UK; 3 Clinical Pharmacology and Safety Sciences, AstraZeneca R&D, Cambridge CB4 0WG, UK; 4 Wellcome Sanger Institute, Wellcome Genome Campus, Hinxton CB10 1SA, UK; 5 Cancer Research UK Cambridge Institute, Cambridge CB2 0AW, UK; 6 Cambridge Centre for AI in Medicine, University of Cambridge, Cambridge CB2 0XY, UK; 7 Wellcome-MRC Cambridge Stem Cell Institute, University of Cambridge, Cambridge CB2 0AW, UK; 8 Berlin Institute of Health, Center for Regenerative Therapies, 13353 Berlin, Germany; 9 Max-Planck-Institute for Molecular Genetics, 14195 Berlin, Germany

**Keywords:** autologous and allogeneic immunogenicity, cell therapies, human primary organoids, cholangiocyte, humanized mouse model, transplantation immunology, spatial transcriptomics, NanoSeq

## Abstract

Primary human cells cultured in long-term expandable 3D organoid format have great promise as potential regenerative cellular therapies, but their immunogenicity has not yet been fully characterized. In this study, we use *in vitro* co-cultures and *in vivo* humanized mouse experimental models to examine autologous and allogeneic immune response to human primary cholangiocyte organoids (PCOs) as treatment for bile duct disorders. Our data demonstrate that PCOs upregulate the expression of human leukocyte antigen (HLA)-I and HLA-II in inflammatory conditions. The allogeneic immune response to PCOs is driven by both HLA-I and HLA-II and is substantially ameliorated by donor-recipient HLA matching. While allogeneic cells display evolving stages of immune rejection *in vivo*, autologous PCOs induce a low-level immune infiltration into the graft site possibly influenced by acquired mutations in culture, cell viability, and culture matrix. Our findings have important implications for the design and clinical translation of autologous and allogeneic organoid cellular therapies.

## Highlights

• Human primary cholangiocyte organoids (PCOs) upregulate HLA-I/II in inflammatory conditions
• Allogeneic immune response is substantially ameliorated by donor-recipient HLA matching
• Autologous PCOs induce a low-level immune infiltration into the graft site
• Humanized mice distinguish early/vigorous and late/resolved stages of allogeneic response

## Graphical abstract

**Figure:**
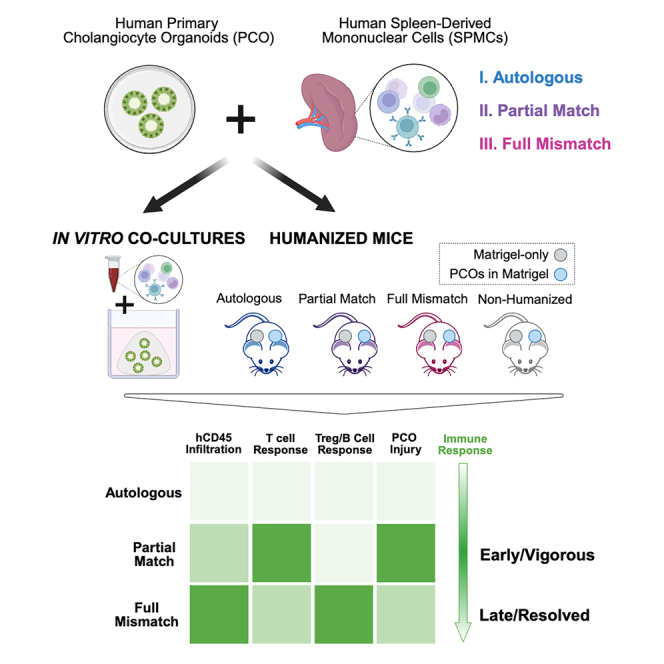

## Teaser

Petrus-Reurer et al. use *in vitro* co-cultures and *in vivo* humanized mice to examine autologous and allogeneic immune response to human primary cholangiocyte organoids (PCOs). The authors show that autologous cells induce a low-level immune infiltration into the graft and allogeneic cells display HLA-driven evolving stages of immune rejection.

## Introduction

Regenerative cellular therapies have emerged as a promising approach for the repair or replacement of diseased or damaged tissues and cells.^1^^,^^2^^,^^3^ The predominant strategy for the generation of therapies is differentiating embryonic stem cells (ESCs) or genetically modified induced pluripotent cells (iPSCs) into the desired cell type.^1^^,^^4^ Cells cultured in 3D organoid format have also recently been developed as an alternative strategy for the generation of cellular therapies.^5^^,^^6^^,^^7^ An important barrier for the clinical translation of cellular therapies is a thorough understanding of their immunogenicity.^8^^,^^9^ This knowledge is critical for informing the use of the clinical strategies for the reduction of the immune response after transplantation, including the use of immunosuppressive drugs, donor-recipient matching, gene editing, and encapsulation of the cells.^10^

It is generally expected that “autologous” cellular therapies, derived from cells obtained from the intended recipient, are unlikely to induce an immune response. However, early studies suggested that syngeneic (autologous) mouse iPSCs may or may not be immunogenic^11^^,^^12^^,^^13^^,^^14^^,^^15^^,^^16^ and that immunogenicity of autologous iPSC-derived cellular therapies may be dependent on the target cell type and the immune microenvironment.^17^^,^^18^^,^^19^^,^^20^ These conflicting findings may be in part because generation of ESC- and iPSC-derived cellular therapies necessarily involves genetic modification that may affect the immunogenicity of the derivative cells. Furthermore, ESC- and iPSC-derived cells may lack full differentiation into an adult phenotype, which may also impact their immunogenicity.^21^ Additionally, other molecular factors independent of cell origin, but instead derived from *in vitro* culturing (e.g., acquired mutations by passaging, cell viability, and culture matrix), may also have a significant impact in triggering immune responses.

Development of autologous cellular therapies represents significant logistic and economic barriers that are likely to limit their widespread clinical use for the foreseeable future.^4^^,^^22^ “Allogeneic” cellular therapies, derived from a different donor than the intended recipient, are thus being explored as potentially cheaper “off the shelf” treatments.^8^ Clinical data from decades of solid organ transplant experience demonstrate that the immune response to most allogeneic solid organs is driven predominantly by human leukocyte antigen (HLA) molecules. Consequently, when permitted by a large pool of donors and recipients (such as in kidney transplantation), HLA matching between the donor and the recipient is an effective strategy for reducing (but not eliminating) the likelihood of immune-mediated rejection and for prolonging graft survival.^23^^,^^24^^,^^25^ Allogeneic cellular therapies are similarly expected to induce an immune response via direct and indirect allorecognition mechanisms driven in large part by HLA. However, it is important to note that most cellular therapies in advanced stages of development consist of a pure population of a single cell type,^26^^,^^27^^,^^28^ in stark contrast to solid organs that are composed of numerous cell types with diverse phenotypes and functions, including antigen-presenting cells that are highly adapted to driving an immune response.

A key challenge to understanding the immune response to human cellular therapies has been the availability of appropriately refined experimental models and access to primary human tissues. Most studies to date have been performed exclusively *in vitro*, have used mouse-derived cellular therapies transplanted into wild-type immunocompetent mice, or have used ESC- or iPSC-derived cells transplanted into mice reconstituted with an allogenic human immune system.^10^^,^^11^^,^^12^^,^^13^^,^^14^^,^^15^^,^^16^^,^^17^^,^^18^^,^^19^ Therefore, a detailed profiling of the human immune response under different HLA-matching scenarios using relevant humanized models and cutting-edge analytical technologies is critical for the advancement of these therapies.

We have previously shown that human primary cholangiocyte organoids (PCOs) can be derived from human cholangiocytes without genetic editing and with long-term expansion potential for use as cellular and bioengineered therapies for bile duct disorders.^29^^,^^30^^,^^31^ In this study, we use a comprehensive panel of *in vitro* and *in vivo* experimental models to unprecedentedly examine the response of autologous and allogeneic (including Partially Matched and Fully Mismatched) immune cells to human PCOs, as an exemplar human primary organoid cellular therapy. We demonstrate that autologous cells induce a detectable immune response, which may be influenced by new mutations acquired during *in vitro* culture, cell viability, and cell matrix. We also show that the allogeneic immune response to PCOs is driven by the level of HLA mismatch between the donor and recipient, showing evolving stages of immune rejection in a humanized mouse model. Our findings provide high-resolution analysis of immunogenicity of autologous and allogeneic organoid cellular therapies, highlighting important implications for their clinical translation.

## Results

### PCOs upregulate expression of HLA-I and HLA-II in inflammatory conditions

Human PCOs were generated and characterized as described previously from gallbladder or bile duct biopsies^29^^,^^30^^,^^31^ taken from more than 70 HLA-typed deceased transplant organ donors. For this work, we selected one bile duct-derived and long-term expanded PCO line (PCO Line 1), which showed characteristic PCO spheroid morphology (Figure S1A) and expression of specific cholangiocyte markers including *SOX17*, *SOX4*, *TFF2*, *FGF2*, *KRT-7*, and *KRT-19* (Figure S1B), in addition to alkaline phosphatase and gamma-glutamyl transferase enzymatic activity comparable to human primary cholangiocytes (Figures S1C and S1D).

**Figure 1:**
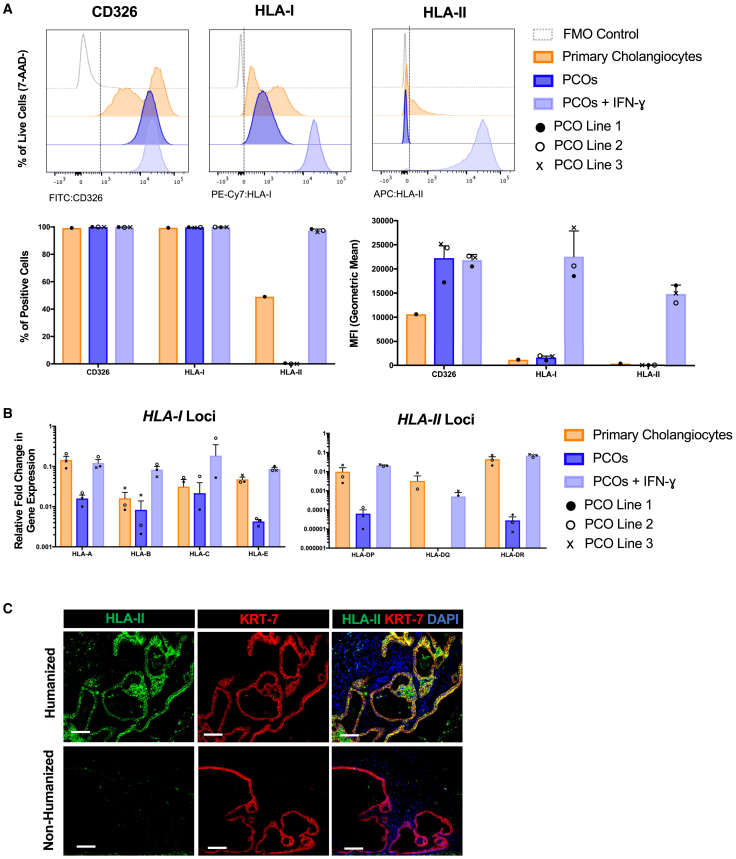
HLA expression by human primary cholangiocytes and PCOs (A) Representative flow cytometry charts (upper row) and bar graphs showing percentage of live cells (7-Aminoactinomycin D [7-AAD] negative) and mean fluorescence intensity (MFI, lower row) of CD326, HLA-I and HLA-II surface expression of human primary cholangiocytes, and PCOs cultured with or without IFN-ɣ stimulation for 2 days from three primary gallbladder/bile duct-derived lines (shown in different symbols). Fluorescence minus one (FMO) controls are used for gating. Error bars represent mean ± SEM from three technical replicates per line in three PCO lines. (B) Quantitative reverse-transcription PCR (RT-qPCR) of *HLA-I* (*HLA-A*, *HLA-B*, *HLA-C*, and *HLA-E*) and *HLA-II* (*HLA-DP*, *HLA-DQ*, and *HLA-DR*) expression by primary cholangiocytes and PCOs cultured with or without IFN-ɣ stimulation for 2 days from three primary gallbladder/bile duct-derived lines (shown in different symbols). Error bars represent mean ± SEM from three technical replicates per lines in three PCO lines. (C) Immunofluorescence images showing expression of human HLA-II and KRT-7 in PCO grafts under the kidney capsule in immunodeficient NSG mice with and without reconstitution with allogeneic human immune cells (humanized). Scale bars: 100 μm. See also Figure S1.

Flow cytometric analysis revealed high bimodal expression of HLA-I and low expression of HLA-II (<40%) by primary cholangiocytes. PCOs cultured under normal conditions abundantly expressed HLA-I but no HLA-II. Both HLA-I and HLA-II were upregulated on PCOs when cultured in inflammatory conditions, simulated by 2 days of co-culture with interferon (IFN)-ɣ. Human primary cholangiocytes and PCOs all expressed the cholangiocyte marker CD326/Epcam as expected (Figure 1A). The relative expression of *HLA-I (A*, *B*, and *C)* and *HLA-II (DP*, *DQ*, and *DR)* genes was quantified using RT-qPCR (Figure 1B), demonstrating upregulation by PCOs in inflammatory conditions for all loci and comparable to primary cholangiocytes. To examine whether the upregulation of HLA-II by PCOs upon co-culture with IFN-ɣ is physiologically relevant, we transplanted PCOs under the kidney capsule of mice reconstituted with an allogeneic human immune compartment (humanized mice). There was robust *in vivo* upregulation of expression of HLA-II by PCOs that were transplanted into humanized mice compared to immunodeficient mice (Figure 1C). These data suggest that both HLA-I and HLA-II are likely to drive the immune response to PCOs in the clinical setting.

### Donor-recipient HLA mismatch determines *in vitro* immune response to PCOs

**Figure 2:**
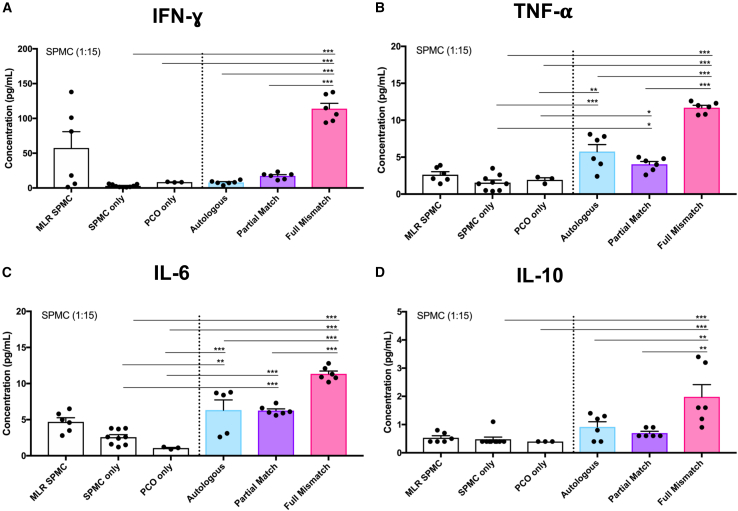
Cytokine secretion of autologous and allogeneic lymphocytes co-cultured with PCOs (A) Bar graphs showing concentration of IFN-ɣ secretion by SPMCs when co-cultured (1:15 ratio) with Autologous, Partial Match, and Full Mismatch PCOs (passage 10) for 5 days. Negative controls are PCOs only and SPMCs only, and positive control is SPMC Mixed Lymphocyte Reaction (MLR) from two different donors. Same conditions were analyzed for TNF-α (B), IL-6 (C), and IL-10 (D). Error bars represent mean ± SEM from three independent experiments with technical duplicates. ∗*p* < 0.0001; ∗∗*p* < 0.001; ∗*p* < 0.01. See also Tables S1, S2, and S3.

We next assessed the level of immune activation as measured by cytokine secretion *in vitro* by co-culturing PCOs with spleen-derived mononuclear cells (SPMCs) derived from same deceased donors used to generate the PCO lines (Table S1), thus enabling immunological experiments with autologous or allogeneic donors with known HLA mismatch. In autologous experiments, PCOs and SPMCs were obtained from the same deceased transplant organ donor. In Partial Match experiments, PCOs and SPMCs were matched at *HLA-I* only (*A*, *B*, and *C* loci). In Full Mismatch experiments, PCOs and SPMCs were mismatched at both *HLA-I* and *HLA-II* loci (*A*, *B*, *C*, *DP*, *DQ*, and *DR*) (Table S2). We used SPMCs (rather than peripheral blood mononuclear cells; PBMCs) for these experiments as it was possible to obtain much larger numbers of SPMCs per donor to enable both *in vitro* and *in vivo* experiments. Of note, we have previously shown that SPMCs can be used for immunological assays *in vitro* and *in vivo.*^32^ Fully Mismatched PCOs induced the strongest and significantly higher immune response as measured by the levels of IFN-ɣ, tumor necrosis factor alpha (TNF-α), and interleukin (IL)-6 secretion compared to other groups (Figures 2A–2C; Table S3). Except for IFN-ɣ, Autologous and Partial Match groups induced a similar level of immune activation that was significantly higher than the negative controls. Note that, interestingly, the levels of IFN-ɣ production induced by Autologous and Partial Match PCOs were not different from negative controls. Secretion of the anti-inflammatory IL-10 was also significantly higher in the Full Mismatch group compared to other groups, potentially suggestive of simultaneous activation of inhibitory pathways (Figure 2D). There were no changes in IL-12p70, IL-13, IL-1β, IL-2, IL-4, and IL-8 secretion levels (Table S3).

### Magnitude of *in vivo* immune infiltration to PCOs is driven by the level of HLA mismatch

**Figure 3:**
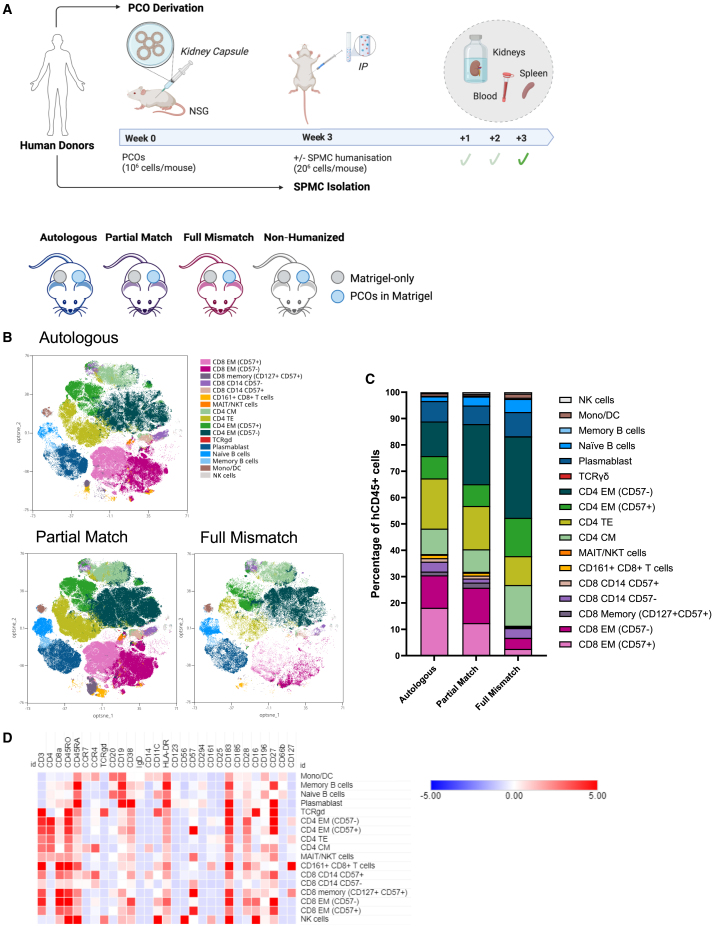
Immune profile of humanized mice transplanted with PCOs (A) (Upper panel) Schematics of the PCO derivation, PCO injection, and subsequent humanization with SPMCs after 3 weeks (*n* = 4–5 per group). Animals were culled, and samples (kidneys, spleens, and blood) collected after 3 weeks of humanization. (Lower panel) Representation of the groups included in the study: PCOs were injected in Matrigel under the left kidney capsule. Matrigel alone was injected under the contralateral kidney capsule to control for Matrigel-driven immune infiltration. Humanization was performed using Autologous, Partial Match, or Full Mismatched human SPMCs. A Non-Humanized group was added as control. (B) T-Distributed Stochastic Neighbor Embedding (t-SNE) visualization and Flow Self-Organizing Map (FlowSOM) clustering show the immune profile of human CD45^+^ cells with the distribution of immune cell subpopulations in different colors. Mice with the same donor engraftment have been overlaid. (C) Stacked bar graph representing the frequency of immune subsets in each type of engraftment. (D) Heatmap illustrating the level of expression of each marker for each cluster defined by FlowSOM analysis (all groups combined). See also Figure S2 and Tables S1, S2, and S4.

We next examined the *in vivo* immune response to PCOs using a humanized mouse model. PCO fragments were injected under the kidney capsule of immunodeficient non-obese diabetic severe combined immunodeficiency gamma (NSG) mice. At 3 weeks post-injection, animals were humanized by intraperitoneal injection of Autologous, Partial Match, or Full Mismatch SPMCs obtained from the same donors as used for the *in vitro* co-culture experiments. Importantly, in each animal, one kidney was transplanted with PCOs in Matrigel, and the contralateral kidney was injected with Matrigel only, thus serving as control for the effect of surgery and possible immune response to the Matrigel matrix. 6 weeks post-injection, animals were culled and their spleens were recovered to assess the human immune cell composition (Figure 3A). Flow cytometry analysis demonstrated 20%–40% human CD45^+^ cells in the spleen, confirming successful humanization (Figures S2A and S2B; Table S4). Cytomety by Time-Of-Flight (CyTOF) analysis revealed reconstitution with predominantly lymphoid cells as expected, with paucity of myeloid cells. Of note, the immune profile in the spleen of the mice was different in animals transplanted with Autologous, Partial Match, or Full Mismatch PCOs (Figures 3B–3D; Table S4). The Full Mismatch group had a decreased abundance of CD8 and a relatively higher proportion of B cells and CD57^−^ effector memory CD4 T cells. Original donor SPMCs also showed differences in abundance of immune cell types (CD4, CD8, natural killer [NK], monocytes/dendritic cells [DC], and B cells), but with overall good representation of all cell types in all groups (Figure S2C; Table S4).

**Figure 4:**
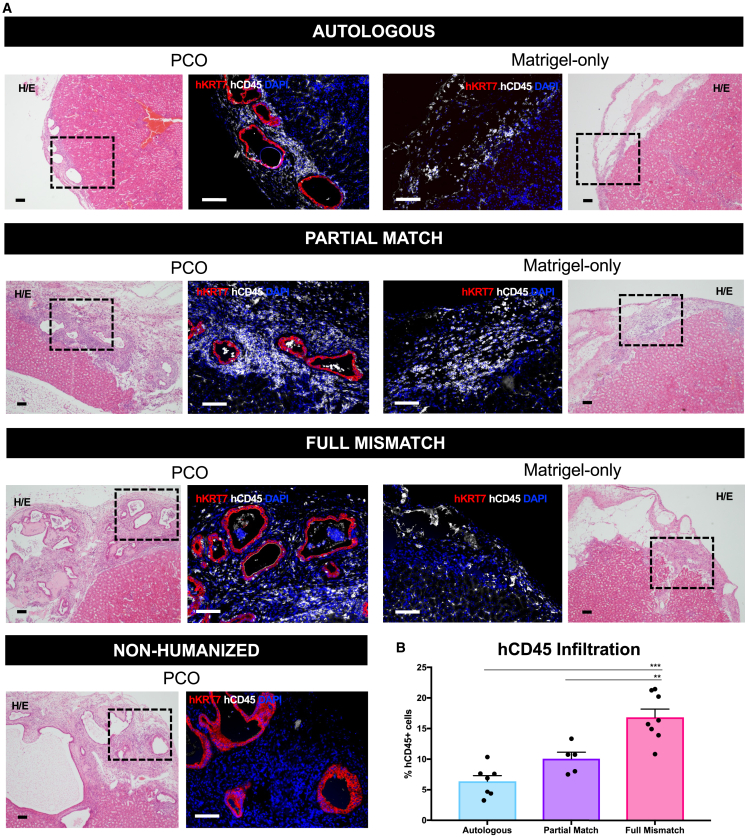
Infiltration of hCD45+ cells into PCO graft sites (A) Hematoxylin/eosin and immunofluorescence images of injected PCOs (passage 10) under the kidney capsule showing expression of human KRT-7 and human CD45 markers in Autologous, Partial Match, Full Mismatch, and Non-Humanized groups. Matrigel-only images, from the same animals, are shown as controls for background immune infiltration induced by surgical procedure and injection of Matrigel. Note that, despite the dense CD45^+^ cell infiltration into the graft site in the Partial Match representative image shown (PCO), this was not induced by the cholangiocytes present in the graft given the similarly dense infiltration also observed in the Matrigel-only control from the contralateral kidney of the same animal. Scale bars: 100 μm. (B) Percentage of hCD45+ cells infiltrating into the graft site quantified in each group relative to the respective Matrigel-only controls (by subtraction). Error bars represent mean ± SEM from 1 to 4 quantified areas per mouse per group. ∗∗∗*p* < 0.0001; ∗∗*p* < 0.01. See also Figures S3 and S4 and Table S5.

Assessment of local immune infiltration to the graft site was performed by immunofluorescence staining with specific human immune and cholangiocytes markers (CD45 and Keratin-7 [KRT-7], respectively). PCO grafts had the characteristic morphology and KRT-7 surface marker expression in all groups (Figure 4A), including upregulation of HLA-II, indicative of alive PCOs responding to surrounding inflammatory response (Figure S3). The entire engrafted areas were scanned, and hCD45+ cells were segmented and counted with a custom-made pipeline (Figure S4; Table S5). The “background” hCD45+ immune infiltration in the Matrigel-only area of the contralateral control kidney was subtracted to correct for non-PCO-driven immune infiltration. The total PCO-specific immune infiltration was significantly greater in the Full Mismatch group, followed by Partial Match and Autologous groups (Figure 4B). Specifically, hCD45+ immune infiltration was 55.3% ± 2.9% in the Autologous group (respective Matrigel-only: 47.9% ± 3.1%), 30.1% ± 1.6% in the Partial Match group (respective Matrigel-only: 20.1% ± 2.6%), and 30.9% ± 2.7% in the Full Mismatch group (respective Matrigel-only: 14.1% ± 5.4%) (Table S5). Importantly, autologous PCOs induced an immune infiltration (5%–10% of total hCD45+ cells) above that seen in the contralateral Matrigel-only control kidneys from the same mice. This apparent immune response to autologous cells was consistent with the low-level immune activation suggested by the *in vitro* co-cultures (Figure 2).

### HLA mismatch determines the phenotype and stage of immune response into the graft site *in vivo*

**Figure 5:**
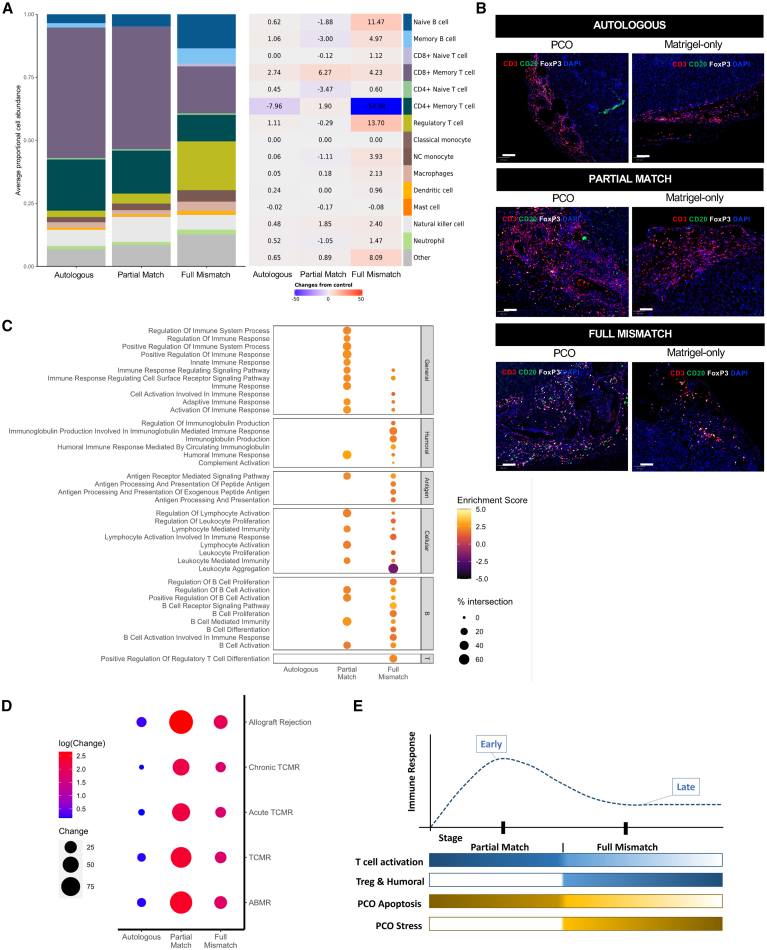
Phenotype of infiltrating immune cells into PCO graft sites (A) Histogram showing abundance of different immune cells types infiltrating into PCO graft sites (left) and their respective fold change compared to Matrigel-only controls (right). Matrix (SafeTME) was extracted from Danaher et al.^33^ (B) Immunofluorescence images of injected PCOs (passage 10) under the kidney capsule for human immune markers CD3, CD20, and FoxP3 in Autologous, Partial Match, and Full Mismatch groups. Matrigel-only images, from the same animals, are shown as controls for background immune infiltration induced by surgical procedure and injection of Matrigel. Scale bars: 100 μm. (C) Pathway enrichment analysis of Autologous, Partial Match, and Full Mismatch groups for general, humoral, antigen, cellular, B cell, and Treg pathways. (D) Dot plot showing the association of Autologous, Partial Match, and Full Mismatch groups with rejection categories extracted from human kidney rejection datasets (TCMR, ABMR, chronic TCMR, acute TCMR, and allograft rejection). (E) Schematic of the evolution of the stages of the human immune response to PCOs with different levels of matching in a humanized mouse model. See also Figures S5 and S6 and Table S6.

To determine the phenotype of the immune infiltrate into the graft site, we performed spatial transcriptomics using the GeoMx platform (NanoString, Bruker), which enabled the retrieval of RNA from hCD45+ and hKRT-7+ cells. Deconvolution of the hCD45+ compartment showed that the infiltrate in the Full Mismatch group had a decrease in CD4^+^ memory T cells and an increase in T regulatory cells (Tregs), B cells (naive and memory), and monocytes relative to Matrigel-only controls (Figures 5A, 5B, and S5A). Difference in immune composition was less prominent between the Autologous and Partial Match groups; however, the former showed a shift from CD4 memory to CD8 memory; and the latter a change from CD4 native to CD8 memory. Overall, there was limited overlap in up-regulated or down-regulated genes between the groups, and different immune-related pathways were enriched in the three groups (Figures 5C, S5B, and S5C; Table S6). In particular, hCD45+ cells in the Autologous group did not show enrichment in any particular immune responses compared to controls (Matrigel-only). Conversely, the Partial Match group depicted general cellular immune activation, while the Full Mismatch group showed an antigen-driven, humoral response in addition to B cell and Treg pathways (Figures 5B and 5C), which is consistent with the cell types described by the deconvolution. Interestingly, the hKRT-7+ compartment shared significantly more up- and down-regulated genes between groups (Figures S6A and S6B; Table S6). Pathway enrichment analysis suggested that PCOs in the Partial Match group had upregulated pathways related to cell death state (e.g., apoptotic pathways), while Full Mismatch PCOs had upregulated cell stress (but not death; e.g., ubiquitin) pathways (Figure S6C). Collectively, the data are consistent with the hypothesis that immune-related pathways are relatively quiescent in the Autologous group but that there is ongoing activation of the immune pathways in the Partial Match and the Full Mismatch groups that have evolved to different stages of the immune response.

### Immune pathways upregulated in humanized mice correlate with those activated by human organs undergoing rejection in transplant recipients

Our data suggest that, compared to the Partial Match group, the immune response in the Full Mismatch group is at a later stage of its evolution, with a dominance of B and regulatory T cells infiltrating the graft site and activation of the associated pathways. Conversely, Partial Match grafts appeared to have induced an earlier “active” stage of the immune response. To examine this hypothesis, we assessed how the patterns of immune activation in the humanized mice compared to those seen in human transplant recipients of solid organs with biopsy-proven active clinical rejection. We thus compared the spatial transcriptomics data from the humanized mice to publicly available human datasets: two kidney rejection microarray datasets^34^^,^^35^; Banff-Human Organ Transplant (B-HOT) dataset^36^ with classified T cell-mediated rejection (TCMR), antibody-mediated rejection (ABMR), acute TCMR, and chronic TCMR; and allogeneic rejection pathways extracted from Kyoto Encyclopedia of Genes and Genomes (KEGG) and PathCards (allograft rejection). Consistent with our hypothesis, we noted that the gene expression profile in human kidneys undergoing rejection correlated best with the Partial Match group and less so with the Full Mismatch group (Figure 5D). Overall, spatial transcriptomics in this SPMC-humanized mouse model captured at a single snapshot in time (6 weeks post-cell therapy injection) suggest that Partial Match immune response corresponds to early stages of response with potent T cell activation and with PCOs showing transcriptional signs of apoptosis. Conversely, Full Mismatch induced an immune response that has evolved to a later stage involving B cells, Tregs, and humoral response, with PCOs showing more transcriptional signs of stress than apoptosis (Figure 5E).

### Mutational burden, viability, and culture matrix composition influence autologous PCO response

**Figure 6:**
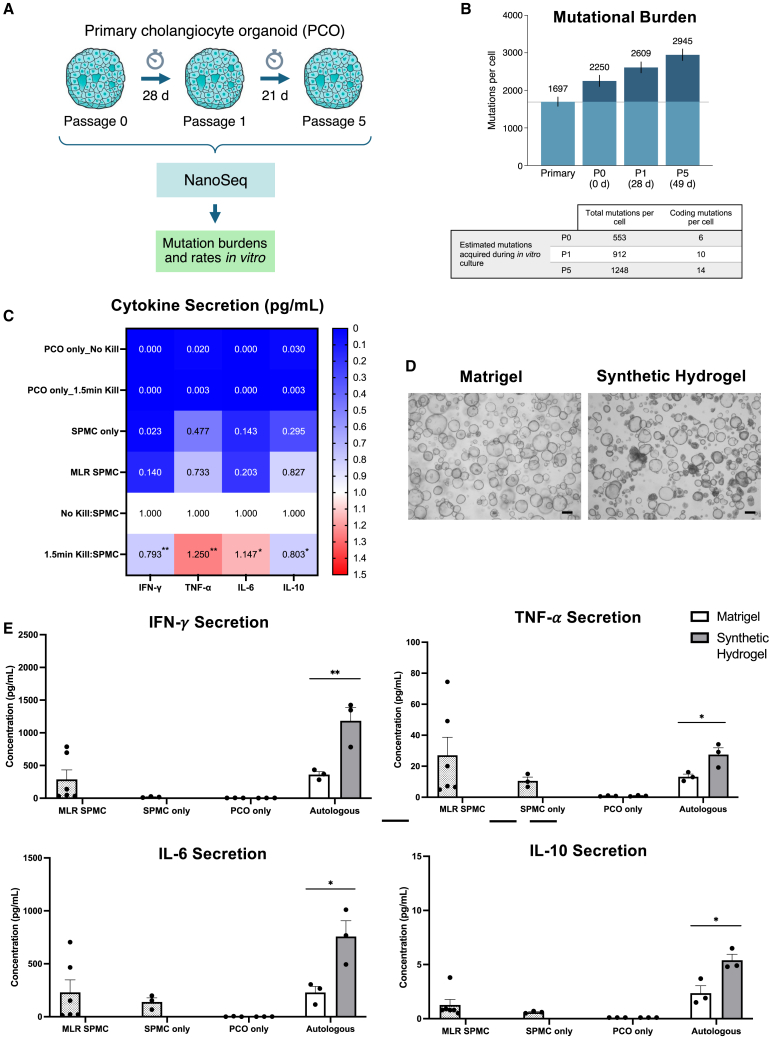
Potential conditions influencing immune response to autologous PCOs *in vitro* (A) Schematic diagram of the experimental design for PCO culture and sequencing. (B) Mutation burdens per cell as estimated through NanoSeq in the primary tissue and PCO samples. Mean burdens per cell are indicated above each bar, with error bars denoting 95% confidence intervals. Mutations in excess of the mutation burden for the primary tissue sample (horizontal line) are colored in dark blue. Passage number (P) and days in culture (d) are indicated for each PCO sample (top). Estimated numbers of mutations acquired during *in vitro* culture for each PCO sample (P0, P1, and P5). Total mutations per cell are calculated as the difference between the mean burdens per cell for the PCO sample and the primary tissue sample (dark blue portion of the bars in B). Coding mutations per cell are calculated by scaling the total mutations per cell by the ratio between whole genome size and protein-coding genome size (bottom). (C) Heatmap showing cytokine secretion of IFN-ɣ, TNF-α, IL-6, and IL-10 by SPMCs when co-cultured (1:15 ratio) with autologous PCOs in normal or killed (80°C, 1.5 min) conditions for 5 days. Values are normalized to No Kill:SPMC co-culture condition. Negative controls are PCOs only and SPMCs only, and positive control is SPMC Mixed Lymphocyte Reaction (MLR). ∗∗*p* < 0.01; ∗*p* < 0.05. (D) Bright-field pictures showing PCOs cultured in Matrigel or synthetic hydrogel for 3 days prior to assay. Scale bars: 200 μm. (E) Bar graphs showing concentration of IFN-ɣ, TNF-α, IL-6, and IL-10 secretion by SPMCs when co-cultured (1:15 ratio) with autologous PCOs for 5 days. Negative controls are PCOs only and SPMCs only, and positive control is SPMC MLR. Error bars represent mean ± SEM from three independent experiments. ∗∗*p* < 0.01; ∗*p* < 0.05.

We next examined potential mechanisms by which autologous PCOs can induce an immune response. To assess the extent of *in vitro* culture-related mutations in PCOs, we sequenced the genomes of organoids at different passages from our PCO line using nanorate sequencing (NanoSeq),^37^ an advanced single-molecule duplex sequencing method allowing ultra-accurate detection of mutations in polyclonal cell populations (Figure 6A). Estimates of mutation burdens per cell for each PCO sample provided evidence for roughly constant acquisition of mutations during culture (Figure 6B). Organoids acquired an average of 1,248 mutations per cell after five passages (49 days), with an estimated average of ∼14 new protein-coding mutations per cell (Figure 6B). This observation is consistent with previous studies reporting accumulation of *in vitro* DNA damage during cell culture in other systems.^38^^,^^39^^,^^40^ Because at least some of these mutations may lead to changes in immunogenic epitopes, it is plausible that some of the immune response to autologous cells could be related to presentation of neoantigens by autologous PCOs.

We also hypothesized that cell viability could impact the immune response to autologous PCOs. To test this, control PCOs (82% viable) and PCOs warmed to 80°C for 1.5 min to induce cell death (40% viable) were co-cultured with autologous SPMCs. The cytokine secretion profile was significantly impacted by the level of viable PCOs in the co-cultures (Figure 6C), therefore indicating that as cells die they may release and express a variety of molecular factors that influence immune response.

Finally, we sought to investigate the impact of the cell culture matrix. PCOs grown in regular Matrigel or in a commercially available synthetic hydrogel (chemically defined) were co-cultured with autologous SMPCs (Figure 6D). As hypothesized, cytokine secretion profile was significantly impacted by the cell matrix composition in which PCOs were cultured in (Figure 6E). Specifically, PCOs in the test synthetic hydrogel triggered a stronger immune response, possibly due to altered cell growth, phenotype, and antigen expression. Overall, our data suggest that *in vitro* culture, cell viability, and cell culture matrix can affect immune response to autologous cells.

## Discussion

This study provides a distinctive and comprehensive comparison of *in vitro* and *in vivo* immune responses to autologous and allogeneic long-term expanded healthy organoids derived from human primary cells. We selected PCOs as our exemplar genetically unedited organoid cellular therapy, as they have previously been shown to have potential utility as a cellular therapy.^29^^,^^30^^,^^31^ We show that human PCOs upregulate HLA-I and HLA-II molecules in inflammatory conditions *in vitro* and *in vivo*. The immunogenicity of PCOs *in vitro*, as quantified by release of inflammatory cytokines by co-cultured lymphocytes, was dependent on the level of donor-recipient HLA matching. Consistent with this finding, immune infiltration into the graft site in humanized mice was also dependent on the level of donor-recipient HLA matching. Autologous PCOs also induced low-level immune infiltration into the graft site compared to controls, maybe influenced by new mutations acquired during *in vitro* culturing, cell viability, and the cell culture matrix. Spatial transcriptomics data indicate evolving stages of immune rejection dependent on donor-recipient HLA matching. Specifically, the immune infiltrate profile in the Partial Match graft sites was consistent with an early and prominent T cell-driven response, compared to a later-stage immune response driven by B cells and Tregs in the Full Mismatch group. Finally, we were able to correlate the immune profile of the graft-infiltrating cells with previously published human kidney allograft rejection profiles.

Here, we focused on the impact of HLA on immunogenicity of the PCOs, because decades of evidence from solid organ transplantation have established HLA as the predominant driver of the alloimmune response and demonstrated the beneficial impact of donor-recipient HLA matching on graft immune rejection and survival.^23^^,^^24^^,^^25^ To enable this study, we obtained paired human primary immune cells and primary cholangiocytes from numerous deceased transplant organ donors. By obtaining large numbers of spleen-derived lymphocytes from every donor, we were able to replicate the same experiments *in vitro* and *in vivo*. Thus, unprecedentedly in a human primary-derived organoid system, we were able to compare the immune response to autologous PCOs, PCOs matched at *HLA-I* (*A*, *B*, and *C*) but mismatched at *HLA-II* (*DP*, *DQ*, and *DR*) loci (Partial Match group), and PCOs mismatched at both *HLA-I* and *HLA-II* loci (*A*, *B*, *C*, *DP*, *DQ*, and *DR*).

It is noteworthy that previous studies assessing immunogenicity of human organoids have either used aggregated non-expandable primary cells^41^ or organoids derived from pluripotent stem cells (thus being substantially genetically modified to differentiate them into [not-fully] functional cells).^42^^,^^43^ The PCOs we present in this study were human primary-derived cholangiocyte cells with comparable 3D morphology and function to primary tissue and with very high expansion potential. It cannot be assumed, therefore, that they will elicit the same immune responses as non-expanding cells or organoids derived from genetically modified pluripotent cells.

Our data demonstrate that PCOs, similar to human primary cholangiocytes, upregulate both HLA-I (*A*, *B*, and *C*) and HLA-II (*DP*, *DQ*, and *DR*) in inflammatory conditions *in vitro* and *in vivo*. Consistent with our findings, upregulation of HLA in inflammatory *in vitro* conditions has been previously reported for other putative cellular therapies derived from ESCs or iPSCs.^44^^,^^45^^,^^46^^,^^47^ These experiments confirm that PCOs have the capacity to induce immune responses to both HLA-I and HLA-II after transplantation (i.e., inflammatory conditions), even if they do not express high levels of HLA-II under ideal culture conditions. The magnitude of the *in vitro* and *in vivo* allogeneic immune responses (as measured by the total number of hCD45+ infiltrating immune cells) was significantly reduced by donor-recipient matching at HLA-I but was nonetheless greater than autologous response, thus being driven by mismatch at HLA-II. These findings are indicative that allogeneic PCOs would induce both anti-HLA-I and anti-HLA-II immune responses in a clinical setting and that donor-recipient HLA matching, at both *HLA-I* and *HLA-II* loci, would be an effective strategy for amelioration of the alloimmune response in patients.

We performed engraftment of the cholangiocyte organoids first and then humanization, to (1) ensure we have surviving graft cells and (2) allow time for resorption of the hydrogel and recovery from surgery, before introducing the immune system. This approach also maximizes the experimental window by minimizing the likelihood of graft vs. host disease in the model. Additionally, by injecting Matrigel alone under the contralateral kidney capsule in every humanized mouse, we controlled for both the immune response induced by surgery and Matrigel itself and the inter-animal variance in immune engraftment that is inevitable with humanized mouse models.^48^^,^^49^^,^^50^ Remarkably, the immune profile of the lymphocytes infiltrating into the graft site *in vivo* was different depending on the level of HLA mismatch. Given that initial immune subsets from original donors have similar composition, and levels of humanization at endpoint were around 60–70% in all analyzed mice, the only difference between study groups resides in the level of HLA matching in the PCOs. This confirms an association between local immune cell infiltration and HLA mismatch of the grafts. Our findings are consistent with the clinical observation that donor-recipient HLA mismatch influences likelihood of immune rejection in liver transplant recipients^51^ and the known mechanisms of allorecognition including the role of HLA in orchestrating T and B cell responses.^52^

Spatial transcriptomics analysis revealed differences in immune cell populations and cell enrichment pathways present at the graft site depending on the level of HLA mismatch. The immune infiltrate in the Partial Match group was dominated by CD8^+^ and CD4^+^ (CD3^+^) T cells, but with B cells and Tregs in the Full Mismatch group. The adaptive immune response to alloantigens is known to evolve over time after transplantation, being initially dominated by effective CD4^+^ and CD8^+^ (CD3^+^) T cells, while at later time points there is a gradual emergence of B cell responses as well as regulatory T cells.^52^^,^^53^^,^^54^ Our findings are consistent with this view of the kinetics of the alloimmune response and suggest the dominance of two different phases of immune response, possibly corresponding to an early/prominent vs. a late/resolved response, in Partial Match and Full Mismatch groups, respectively. In fact, the prominent immune profile captured in the Partial Match group was highly associated with human rejection signatures extracted from datasets of human kidneys. Although PCO grafts for all groups demonstrated characteristic morphology and maker expression (KRT-7+) as well as expected response to an inflammatory milieu (HLA-II upregulation), spatial transcriptomics suggested a more apoptotic-like state in the Partial Match group. These results are consistent with an evolving immune response, comprising a more vigorous early immune response captured in the Partial Match group and a later quiescent stage in the Full Mismatch group.

Interestingly, we also observed greater production of IL-10, a predominantly anti-inflammatory cytokine, in lymphocyte *in vitro* co-cultures with Full Mismatch PCOs. Release of IL-10 and other inhibitory cytokines has indeed been postulated as one of the negative feedback mechanisms to curtail ongoing immune responses to alloantigens, including from NK cells.^55^ However, we were unable to experimentally test how donor-recipient HLA mismatch influences the kinetics of the evolution of the immune response to PCOs, because all animals were culled at a single fixed time point after immune reconstitution. It is also possible that differences in the donor immune compartment or immune engraftment in the animals contributed to some of the differences in the immune responses captured in our study. Of note, however, the composition of the immune compartment of the donors was broadly similar.

The immune response to autologous human cellular therapies has been reported in previous studies with conflicting findings.^17^^,^^18^^,^^19^^,^^56^^,^^57^^,^^58^ Our data suggest that autologous PCOs may also induce a low-level immune response. Autologous PCOs induced *in vitro* production of higher levels of the inflammatory cytokines TNF-α and IL-6, but not IFN-ɣ. Autologous PCOs also induced greater infiltration of immune cells into the graft site compared to control (Matrigel-only) injection sites. Following up on previous studies demonstrating the mutational impact of *in vitro* culturing,^59^^,^^60^ we hypothesized that some low-level autologous response could be caused by mutations acquired with PCO culture. Here we show that mutations are acquired by passage, with a proportion affecting coding regions, which could plausibly lead to the expression of neoantigens and drive immune response in *in vitro*-expanded autologous cells. Additionally, we interrogated if cell viability (inevitable when cells are cultured *in vitro* and after manipulation for transplantation *in vivo*) and cell matrix composition could influence autologous immune response. Our results indicate that autologous co-cultures are significantly impacted by both cell viability and culture matrix, and possibly other factors. Although our data confirm that culture conditions affect expression of HLA on PCOs, the specific molecules and factors that are up- or down-regulated to induce these responses are yet to be identified. Despite this, it is not possible to definitively conclude whether autologous PCOs would induce a similar immune response in a clinical setting or if such weak immunogenicity would be clinically relevant and lead to significant cell loss or require short-term immunosuppression. Overall, these analyses allude to the importance of characterizing the immunogenicity of any culture prior to its use as cellular therapy at both genetic and phenotypic levels despite their autologous origin.

In summary, our study confirms that both HLA-I and HLA-II are likely to drive T and B cell-dependent immune response to organoid cellular therapies in a clinical setting. Donor-recipient HLA matching and elimination or HLA expression by genetic editing are likely to be effective strategies to substantially ameliorate, but not eliminate, the immune response to allogeneic cellular therapies in a clinical setting. This is consistent with transplant studies that have identified minor histocompatibility (and other) molecules as making a small contribution to the alloimmune response.^61^ Our findings suggest that some immunosuppressive therapies are still likely to be required in patients receiving optimally HLA-matched cellular therapies. Moreover, our data also do not exclude the possibility that autologous cellular therapies may similarly induce a low-level immune response in patients. However, it is well established that minimizing the overall burden of immunosuppression reduces its adverse effects.^62^ Elimination of the alloimmune response against HLA-I and HLA-II, through the use of autologous cellular therapies, HLA-matching, or genetic editing, would thus be expected to substantially reduce the required dose of immunosuppression and associated adverse effects in patients. Taken together, this work provides a high-resolution perspective to guide future efforts focused on deeper understanding of the immunogenicity of cellular therapies and help accelerate the clinical translation of cell-based products for regenerative medicine applications.

### Limitations of the study

In our *in vivo* experiments, we transplanted PCOs under the kidney capsule rather than into the liver. We chose the kidney capsule because it is a highly vascular niche^63^^,^^64^ that supports the survival of PCOs, enables localization of the graft at the experimental endpoint, and represents a well-characterized niche for assessment of immunogenicity. It is possible, however, that the immune response to PCOs may differ qualitatively or quantitatively from our findings when transplanted into the liver in patients. While we used unselected SPMCs (consisting of both lymphoid and myeloid cells) in our *in vitro* experiments, our humanized mouse models primarily recapitulated the lymphoid human immune compartment. It is thus possible that recipient myeloid cells may also influence the immune response to PCOs in a clinical setting. Future studies utilizing other experimental models would be necessary to examine the contribution of myeloid cells to the alloimmune response to PCOs. Notwithstanding these caveats, it is important to note that our *in vitro* and *in vivo* experimental models were sufficiently refined to distinguish between the immune response to allogeneic PCOs with different levels of donor-recipient HLA mismatch.

## Resource availability

### Lead contact

Requests for further information should be directed to and will be fulfilled by the lead contact, Kourosh Saeb-Parsy (ks10014@cam.ac.uk).

### Materials availability

Cholangiocyte organoids described in this publication will be made available on request from the University of Cambridge under a materials transfer agreement with the university.

### Data and code availability

• Raw mass cytometry data are available on Zenodo (https://doi.org/10.5281/zenodo.10054742). Spatial transcriptomics data (FASTQ files, processed count matrices, annotation files, and metadata) are available on the Gene Expression Omnibus (GEO: GSE253679) and Dryad (https://doi.org/10.5061/dryad.g4f4qrfx9). DNA sequencing data are available on the European Genome-phenome Archive (EGA: EGAD00001015456).
• Code for spatial transcriptomics analyses has been deposited on Zenodo (https://doi.org/10.5281/zenodo.10054742). Custom computer code for DNA sequencing data has been deposited on GitHub (github.com/baezortega/PCO2024).
• Any additional information required to reanalyze the data reported in this paper is available from the lead contact upon request.

## Acknowledgments

We are grateful to the donors and their families and the Cambridge Biorepository for Translational Medicine (CBTM) for the previous gifts of tissue donation. We would also like to thank Jasper Callemeyn and Maarten Naesens for their input on the analysis of human kidney transplantation datasets and Fotios Sampaziotis for his input into the generation of cholangiocyte organoids. Figures were created in GraphPad Prism 10. The graphical abstract and Figure 3A drawings were created with BioRender.com.

S.P.-R. was supported by awards from the Medical Research Council (10.13039/501100000265MRC) 10.13039/501100019326UK Regenerative Medicine Platform (MR/S020934/1) and 10.13039/501100000265MRC Confidence in Concept (G116517). I.M. is funded by 10.13039/501100000289Cancer Research UK (C57387/A21777), the Doctor Josef Steiner 10.13039/100002002Cancer Foundation and the core funding from Wellcome Sanger Institute (220540/Z/20/A). L.V. was supported by the 10.13039/501100000781European Research Council grant New-Chol award (ERC: 741707).

## Author contributions

S.P.-R. conceived and designed the study; performed experiments; acquired, interpreted, and analyzed the data; and drafted and edited the manuscript. W.L. performed bioinformatic analysis. O.T. conceived and designed the study, generated PCO lines, and performed experiments. M.M. and C.B. performed CyTOF experiments and analyzed data. A.B.-O. performed bioinformatic mutational analyses. T.T. and S.R. contributed to tissue sectioning experiments. K.T.M. provided human primary tissue and isolated splenocytes. J.J. and C.B. performed slide scanning and hCD45 quantification. N.H. provided funding and supported data analysis. I.M. provided funding and interpreted mutational data. L.V. provided funding and supported the generation of primary cholangiocyte organoids. K.S.-P. conceived and designed the study, performed animal experiments, interpreted data, and wrote and edited the manuscript. All authors approved the manuscript.

## Declaration of interests

K.S.-P. and L.V. are founders and shareholders of Bilitech Ltd and inventors on the patent applications GB/19.96.17 and GBA 201709704 held by the University of Cambridge that cover the derivation and use of cholangiocyte organoids for regenerative medicine.

## STAR★Methods

### Key resources table

**Table:** 

REAGENT or RESOURCE	SOURCE	IDENTIFIER
**Antibodies**		

Mouse monoclonal HLA-ABC-PeCy7, clone [G46-2.6], 1:20	Biosciences	CAT# 561349; RRID:AB_10612559
Mouse monoclonal HLA-DR, DP, DQ-APC, clone [Tü39], 1:20	Biolegend	CAT# 361714; RRID:AB_2750316
Mouse monoclonal CD326-FITC, clone [9C4], 1:20	Biolegend	CAT# 324204; RRID:AB_756078
Mouse monoclonal CD45-FITC, clone [HI30], 1:50	Thermo Fisher Scientific	CAT# 11-0459-42; RRID:AB_10852703
Mouse monoclonal CD45.1-PE-Cy7, clone [A20], 1:50	Thermo Fisher Scientific	CAT# 25-0453-82; RRID:AB_469629
Mouse monoclonal CD45, clone [HI30], 1:100	BD Biosciences	CAT# 555480; RRID:AB_395872
Rabbit monoclonal KRT-7, clone [EPR1619Y] 1:200	Abcam	CAT# ab68459; RRID:AB_1139824
Donkey anti-rabbit IgG (H+L) Alexa Fluor 555, 1:200	Thermo Fisher Scientific	CAT# A31572; RRID:AB_162543
Donkey anti-mouse IgG (H+L) Alexa Fluor 647, 1:200	Thermo Fisher Scientific	CAT# A31571; RRID:AB_162542
Donkey anti-mouse IgG (H+L) Alexa Fluor 488, 1:200	Thermo Fisher Scientific	CAT# A21202; RRID:AB_141607
Mouse monoclonal CD45 antibody clone [2B11 + PD7/26], 1:250	Agilent	CAT# M0701; RRID:AB_2314143
Mouse monoclonal KRT-7, clone [KRT7/760], 1:200	Abcam	CAT# ab215855
Mouse monoclonal CD3, clone [UMAB54], 1:100	Origene	CAT# UM000048BF
Rabbit monocloncal CD45, clone [D9M8I], 1:200	CST	CAT# 13917BF
Mouse monoclonal HLA-DP, DQ, DR, clone [CR3/43], 1:100	Agilent	CAT# M0775; RRID:AB_2313661

**Biological samples**		

Primary biliary and spleen tissues isolated from deceased transplant organ donors	UK Hospitals	N/A
Kidney xenograft tissue	This paper	N/A

**Chemicals, peptides, and recombinant proteins**		

Matrigel	Corning	356237
Red Cell Lysis Buffer	Stem Cell Technologies	07850
William’s E Medium	Thermo Fisher Scientific	A1217601
human Dickkopf-related Protein (hDKK)	R&D Systems	5439-DK-01M/CF
human Epidermal Growth Factor (hEGF)	R&D Systems	236-EG-01M
hR-Spondin-1	R&D Systems	4645-RS/CF
Rock Inhibitor-Y27632	Selleckchem	S1049
RPMI 1640 Medium	Thermo Fisher Scientific	11875093
TrueGel3D Hydrogel Kit	Sigma-Aldrich	TRUE1
Accutase	Sigma-Aldrich	A6964
DNAse-I	Sigma-Aldrich	4536282001
Fetal Bovine Serum (FBS)	Thermo Fisher Scientific	10082-147
Ethylenediaminetetraacetic Acid (EDTA)	Thermo Fisher Scientific	15575-020
Phosphate Buffer Saline (DPBS)	Sigma-Aldrich	D8537
7-Aminoactinomycin D (7-AAD)	Biolegend	420404
*Dimethyl sulfoxide* (DMSO)	Sigma-Aldrich	D8418-250mL
Lymphoprep	StemCell Technologies	07851
Rnase-free Dnase	Qiagen	79254
RnaseH	Thermo Fisher Scientific	18021071
Random Hexamers	Thermo Fisher Scientific	N8080127
Superscript III Reverse Transcriptase	Thermo Fisher Scientific	18080085
Taq-polymerase	Thermo Fisher Scientific	4304437
Interferon-gamma (IFN-ɣ)	R&D Systems	285-IF-100
AB Serum	Sigma-Aldrich	H3667
Penicillin-Streptomycin	Thermo Fisher Scientific	15140-122
Interleukin-2 (IL-2)	BD Biosciences	554603
CD28	Biolegend	302902 [CD28.2]
Ethylenediaminetetraacetic Acid (EDTA)	Sigma-Aldrich	E5134-500g
Tris Base	Sigma-Aldrich	93352
Tween-20	Sigma-Aldrich	P9416
DAPI-Hoechst 33342	Invitrogen	H3570
Human TruStain FcX	BioLegend	422302
Maxpar Cell Acquisition Solution	Fluidigm	201240
0.1X EQ Four Element Calibration beads	Fluidigm	201078
Maxpar Cell Staining Buffer	Fluidigm	201068
Epitope Retrieval Solution 2 (ER2)	Leica	AR9640
ProLong Diamond Antifade Mountant	Invitrogen	P36970
Syto 83	Thermo Fisher Scientific	S11364
Fluorescence Mounting Medium	Agilent	S3023
Protein Block	DAKO	X090930-2
DAB Enhancer	Leica	AR9432
ProLong Diamond Antifade Mountant	Invitrogen	P36970
DPX Mountant for Histology	Sigma-Aldrich	06522-500ML

**Critical commercial assays**		

10-plex Human Proinflammatory Panel	MesoScale Discovery	K15049D-2
RNeasy Plus Mini Kit	Qiagen	74106
Maxpar Human Immune Monitoring Panel Kit	Fuidigm, CA	201234
Akoya Biosciences Opal 6-Plex Detection Kit	Akoya	NEL871001KT
QIAmp DNA Blood Mini Kit	Qiagen	S1104
Qubit dsDNA (broad range) BR Assay Kit	Invitrogen	Q32850
GenDx NGSgo-MX11-3 Kit	GenDx	7971864
MiSeq Reagent Kit v2	Illumina	MS-102-2002

**Deposited data**		

Raw Mass Cytometry data	This paper	https://doi.org/10.5281/zenodo.10054742
Raw and analyzed data: Spatial Transcriptomics	This paper	GSE253679 https://doi.org/10.5061/dryad.g4f4qrfx9
Raw and analyzed data: DNA sequencing	This paper	EGAD00001015456

**Experimental models: Organisms/strains**		

Mouse: Nod Scid Gamma (NSG)	Charles River	614NSG

**Oligonucleotides**		

Taqman probe for *GAPDH*	Thermo Fisher Scientific	HS02786624_g1
Taqman probe for *HLA-A*	Thermo Fisher Scientific	HS01058806_g1
Taqman probe for *HLA-B*	Thermo Fisher Scientific	HS00818803_g1
Taqman probe for *HLA-C*	Thermo Fisher Scientific	HS00740298_g1
Taqman probe for *HLA-E*	Thermo Fisher Scientific	HS03045171_m1
Taqman probe for *HLA-DP*	Thermo Fisher Scientific	HS00410276_m1
Taqman probe for *HLA-DQ*	Thermo Fisher Scientific	HS03007426_mH
Taqman probe for *HLA-DR*	Thermo Fisher Scientific	HS00219575_m1

**Software and algorithms**		

FlowJo v10	Tree Star	https://www.flowjo.com/
ImageJ v2.0	ImageJ	https://imagej.nih.gov/ij/
GraphPad Prism 10	GraphPad	https://www.graphpad.com/features
QuPath-0.5.1-x64	QuPath	https://qupath.github.io/
HALOImage Analysis Platform version 3.6.4134	Indica Labs, Inc	https://indicalab.com/halo/
HALO AI version 3.6.4134	Indica Labs, Inc	https://indicalab.com/halo-ai/
OMIQ	OMIQ	https://www.omiq.ai/
FlowSom	Bioconductor	https://www.bioconductor.org/packages/release/bioc/html/FlowSOM.html
GenDx NGSengine, version 2.28.1; 3.50 IMGT/HLA release version	GenDx	https://www.gendx.com/product_line/ngsengine/
Python v3.7.4	Python Software Foundation	https://www.python.org
R v4.2.2 and v4.0.2	The R Project for Statistical Computing	https://www.r-project.org/

**Other**		

Custom computer code for spatial transcriptomics analyses	This paper	https://doi.org/10.5281/zenodo.10054742
Custom computer code for spatial transcriptomics analyses DNA sequencing analyses	This paper	github.com/baezortega/PCO2024

### Experimental model and study participant details

#### PCO derivation and passage

Cholangiocyte lines were derived from tissue gifted by deceased transplant organ donors under ethical approval (NRES Committee East of England - Cambridge South, ref. 15/EE/0152) and informed consent from the donor families. Donors were males and females between 46-73 years old (see specifics in Table S1). After retrieval, extrahepatic bile ducts were collected, cut as flat sheets with sterile scalpel, and washed three times in separate tubes with PBS (Sigma, D8537). In a petri dish with cholangiocyte media (CM, see ref.^30^), cells from the lumen were scraped until velvet structure was not visible. Tissues were flushed with CM and all media was collected and centrifuged 5 minutes, 400g. If pellet appeared red, a red cell lysis removal step with ammonium chloride solution (Stem Cell Technologies, 07850) was performed (12 minutes, 4°C). After, cells were washed twice with CM and debris and fibrous tissues pieces were removed with pipette. Plating was performed according to pellet size resuspending cells in 2:3 of Matrigel (Corning, 356237) and 1:3 of complete cholangiocyte media (CCM)^30^ consisting of William’s E medium (Thermo Fisher Scientific, A1217601) supplemented with human Dickkopf-related Protein (hDKK, 100ng/mL (R&D Systems, 5439-DK-01M/CF); human Epidermal Growth Factor (hEGF, 50ng/mL (R&D Systems, 236-EG-01M); hR-Spondin-1 500ng/mL (R&D Systems, 4645-RS/CF) and Rock Inhibitor-Y27632 10mM (Selleckchem, S1049). 50uL-domes were plated in 24-well or 6-well plates kept at 37°C for 3 minutes and subsequently flipped up-side-down at 37°C for 15 minutes. CCM was then added to the well at a final volume of 1mL (24-well) or 3mL (6-well), and cultures were maintained in CCM without Rock Inhibitor-Y27632 in a 5% CO_2_ incubator. Typical PCO morphology appeared after 2-3 days and organoids were ready to passage after 7-10 days.

Established PCO lines were passaged mechanically at 1:3-1:5 ratio every 3-5 days as follows: organoids were retrieved from domes in a collection tube using Cell Recovery Solution (Corning, 354253). Pooled domes in the solution were kept on ice for 15 minutes. Tubes were centrifuged for 5 minutes, 400g and washed with 1mL of CM. Pellet size were assessed and divided in tubes for subsequent passage. PCO pellets were resuspended with 1:3 of CCM and mechanically fragmented with a p200 (40-60 times) until middle-size homogenous fragments were observed under the microscope. Then, 2:3 of Matrigel was added to the fragmented PCOs per tube, resuspended well, and 50μL-domes were plated in 24-well or 6-well plates. Plated domes were kept at 37°C for 3 minutes and subsequently flipped up-side-down at 37°C for 15 minutes. CCM media was then added to the well to a final volume of 1mL (24-well) or 3mL (6-well). Cultures were fed three times a week with CCM without Rock Inhibitor-Y27632 in a 5% CO_2_ incubator.

For the experiments with Synthetic Hydrogel, TRUE1 (TrueGel3D Hydrogel Kits, Sigma-Aldrich) was purchased and prepared according to manufacturer’s instructions. PCO pellets were mechanically fragmented with a p200 (40-60 times) until middle-size homogenous fragments were observed under the microscope. They were then mixed with the respective volume ratio of each hydrogel component (RGD degradable polymer 72%, CD cell-degradable crosslinker 8% and CCM with Rock Inhibitor-Y27632 20%). 50μL-domes were plated in 24-well plate and kept at 37°C for 30 minutes. CCM media was then added to the well to a final volume of 1mL (24-well) and replaced after 1 hour at 37°C. Cultures were fed three times a week with CCM without Rock Inhibitor-Y27632 in a 5% CO_2_ incubator.

#### Mouse model

Fifteen male and female (half-half) immunodeficient Nod Scid Gamma (NSG, 614NSG) mice aged 6-10 weeks (average body weight of 22.4 ± 0.8 g) were obtained from Charles River Labs Co., Ltd (UK) for this animal study. Animal experiments were conducted in accordance with UK Home Office and Institutional regulatory requirements (HO PP5753595). The mice were housed under specific pathogen-free conditions, where they were maintained under controlled environmental conditions (temperature: 22 ± 2 C; humidity: 45% ± 5%) with a 12h light-dark cycle. They were provided with a standard commercial diet and sterile water *ad libitum* throughout the study, in accordance with standard guidelines for animal care and husbandry.

#### PCO injections and humanization

An average of 15 domes of PCOs (passage 10) were used for transplantation into each animal. PCOs were retrieved from domes in a collection tube using Cell Recovery Solution (Corning, 354253). Pooled domes in the solution were kept on ice for 15 minutes. Tubes were centrifuged for 5 minutes at 400g and washed with 1mL of CM. PCO pellets were resuspended with CCM and mechanically fragmented with a p200 pipette (40-60 times) until middle-size homogenous fragments were observed under the microscope. All fragments were combined together and well-resuspended with 1:3 of CCM media and 2:3 of Matrigel (Corning, 356237). Cells were transported on ice and loaded into a 50mL syringe (Hamilton 700/1700 Series Microliter/Gastight Syringes, Model: 1705) with a blunt needle (custom-made, FisherScientific, 10698205). 25μL of cell mix was injected under the kidney capsule of left kidney following a left flank incision to deliver the kidney onto the operating field under isoflurane anaesthesia. Matrigel-only was injected under the right kidney capsule as control. After surgery, animals were administered additional saline and mesh food, and were closely monitored for any adverse effects.

2 weeks after cell injection, animals (n=4-5 per group, randomized) were humanized with 20x10^6^ human SPMCs per mouse. SPMCs were thawed in a water bath at 37°C, washed with 50% Fetal Bovine Serum (FBS) in RPMI 1640 medium (Thermo Fisher Scientific, 11875093) followed by 10% FBS in RPMI wash, and filtered through a 40mm strainer (FisherBrand, 22363547). Cell numbers were assessed by counting with Countess II FL (Life Technologies). Total amount of cells needed were resuspended in total volume needed of 2% FBS in PBS and kept on ice. 200μL of SPMCs were injected intraperitoneally per mouse with a 29g needle (Beckton Dickinson, 324892). Humanization was evaluated weekly by flow cytometry of tail-vein bleeds collected in heparin-coated tubes (Sarstedt Ltd, 20.1309), followed by red cell lysis removal (StemCell Technologies, 07850) according to manufacturer’s instructions and using the following conjugated antibodies: mouse anti-human CD45-FITC (1:50, Thermo Fisher Scientific, 11-0459-42, clone [HI30]) and mouse anti-mouse CD45.1-PE-Cy7 (1:50, Thermo Fisher Scientific, 25-0453-82, clone [A20]). Cells were incubated at 4°C for 30 min and washed twice with FACS Buffer and 7-Aminoactinomycin D (7-AAD) viability staining solution (1:50, Biolegend, 420404) was added to the cells and run with FACS Canto II (BD Biosciences). Analysis of the data was carried out using FlowJo v.10 software (Tree Star). Results are presented as mean±SEM (standard error of the mean).

### Method details

#### Flow cytometry

PCOs were retrieved from domes as described for passaging and pellets were resuspended with 1mL of Accutase (Sigma, A6964), DNAse-I (NEB, Sigma, 4536282001) and Rock Inhibitor-Y27632 10mM (Selleckchem, S1049) per tube. Tubes were placed in a water bath at 37°C for 5 minutes, followed by 10-times gentle resuspension. The single cell suspension was evaluated under microscope until clumps were not seen (if needed, tubes were placed in a water bath 37°C for extra 5 minutes). 3mL of FACS Buffer (2% FBS (Thermo Fisher Scientific, 10082-147) and 1mM Ethylenediaminetetraacetic Acid (EDTA, Thermo Fisher Scientific, 15575-020) diluted in 1x Phosphate Buffer Saline (DPBS, Sigma, D8537) was added to stop the reaction and cells were centrifuged for 5 minutes 400g. Cells were filtered with a 40μM strainer (FisherBrand, 22363547), centrifuged and further stained using the following conjugated antibodies: mouse anti-human HLA-ABC-PeCy7 (1:20, BD Biosciences, 561349, clone [G46-2.6]), HLA-DR, DP, DQ-APC (1:20, Biolegend, 361714, clone [Tü39]) and CD326-FITC (1:20, Biolegend, 324204, clone [9C4]).

Cells were incubated with the conjugated antibodies at 4°C for 30 min and washed twice with FACS Buffer. Fluorescence minus one (FMO) controls were included for each condition to gate negative and positive cells. 7-AAD viability staining solution (1:50, Biolegend, 420404) was added to the cells. Respective Geometric Mean Fluorescence Intensity (GMFI) values of the FMO controls were subtracted to obtain the final GMFI of each fluorophore. Stained cells were analyzed using a FACS Canto II (BD Biosciences). Analysis of the data was carried out using FlowJo v10 software (Tree Star). Biological triplicates were used for every condition. Results are presented as mean±SEM (standard error of the mean).

#### RT-qPCR

Total RNA was isolated using the RNeasy Plus Mini Kit (Qiagen, 74106) and treated with Rnase-free Dnase (Qiagen, 79254) and RnaseH (Thermo Fisher Scientific,18021071) according to manufacturer’s protocol. cDNA was synthesized using 1 μg of total RNA in a 20 μL reaction mixture containing Random Hexamers (Thermo Fisher Scientific, N8080127) and Superscript III Reverse Transcriptase (Thermo Fisher Scientific, 18080085), according to the manufacturer’s instructions.

Taq-polymerase (ThermoFisher Scientific, 4304437) together with Taqman probes (ThermoFisher Scientific) for *GAPDH* (HS02786624_g1), *HLA-A* (HS01058806_g1), *HLA-B* (HS00818803_g1), *HLA-C* (HS00740298_g1), *HLA-E* (HS03045171_m1), *HLA-DP* (HS00410276_m1), *HLA-DQ* (HS03007426_mH), *HLA-DR* (HS00219575_m1) were used. Samples were subjected to real-time PCR amplification protocol on CFX96 Real-Time System (BioRad). Biological triplicates were performed for every condition and technical duplicates were carried out for each reaction. Results are presented as mean±SEM (standard error of the mean).

#### HLA Genotyping

Genomic DNA from cell pellets was isolated using QIAmp DNA Blood Mini Kit (Qiagen, S1104) following manufacturer’s instructions. DNA was quantified and purity assessed using NanoDrop One/OneC Microvolume UV-Vis Spectrophotometer (Thermo Fisher Scientific). Multiplex Human Leukocyte Antigen (HLA) amplification of 11 loci (*HLA-A∗, B∗, C∗, DRB1∗, DRB3/4/5∗, DQA1∗, DQB1∗, DPA1∗* and *DPB1∗*) was performed with GenDx NGSgo-MX11-3 Kits (GenDx, 7971864). The library was quantified using Qubit dsDNA (broad range) BR Assay Kit (Invitrogen, Q32850) and the final library loading concentration was 15pM. Sequencing was performed using MiSeq instrument (Illumina) and MiSeq Reagent Kit v2 (Illumina, MS-102-2002) with 300 cycle flow cells. Analysis was done using GenDx NGSengine software (version 2.28.1; 3.50 IMGT/HLA release version) complying with European Federation of Immunogenetics (EFI) accreditation.

#### SPMC Isolation from human donors

Human spleen-derived mononuclear cells (SPMCs) were isolated from deceased transplant organ donors by cutting a portion of spleen into small pieces with a sterile scalpel, dissociated using the gentleMACS Tissue Dissociator (Miltenyi; programme B), and filtered through a 70μm strainer (Falcon, 352350) with 20mL of cold 2% FCS in PBS. The suspension was slowly layered on top of 15mL of room temperature Lymphoprep (StemCell Technologies, 07851). Cells were centrifuged at 800g for 20 minutes at room temperature (brake off), the mononuclear cell layer was transferred into a new tube washed with 2% FCS in PBS, and further centrifuged at 300g for 7 min. Red cell blood cell lysis buffer (StemCell Technologies, 07850) was applied according to manufacturer’s instructions in case pellets appeared red. Cells were counted with Countess II FL (Life Technologies) and frozen in 10% *Dimethyl sulfoxide* (DMSO, Sigma, D8418-250mL) in FCS accordingly (50 million cells/mL per vial).

#### SPMC Isolation from mouse spleens

Spleens were harvested immediately following the killing of the animals and placed in RPMI on ice until processing. Tissues were dissociated into a single cell suspension for mass cytometry analysis by mechanical breakdown and washing through a 70μm cell strainer with 10% of FCS-RPMI. The resulting cell suspensions were then washed with 20mL of 10% of FCS-RPMI and centrifuged at 300g for 8 minutes. The pellets were resuspended in 1mL of red blood lysis buffer (11814389001, Roche), incubated for 5 minutes at room temperature (RT) prior to wash again with 9mL of serum-free RPMI followed by centrifugation at 300g for 5 min. The pellets were resuspended in 1mL of serum-free RPMI for counting.

#### Co-culture of PCOs with SPMCs

SPMCs were first thawed in a water bath 37°C, washed with 50% FBS in RPMI followed by 10% FBS in RPMI wash, and filtered through a 40mm strainer (FisherBrand, 22363547). Cell numbers were assessed by counting with Countess II FL (Life Technologies). 2 days Interferon-gamma (IFN-ɣ) pre-stimulated (100 ng/mL, R&D Systems, 285-IF-100) PCOs (passage 10) were single celled as described above and plated at a cell density range of 9x10^5^ (1:15) cells/cm^2^ using a 1:1 mix of CCM media and RPMI medium (Thermo Fisher Scientific, 21875-034) with 10% AB serum (Sigma, H3667) and 100U/mL Penicillin-Streptomycin (Thermo Fisher Scientific, 15140-122). 1.5x10^6^ of isolated SPMCs were plated per well in a 48-well plate on top of the plated PCOs. Interleukin-2 (IL-2, 1 ng or 100U, BD Biosciences, 554603), CD28 (1.25 μg/mL, Biolegend, 302902 [CD28.2]) molecules were added to the wells. For Mixed Lymphocyte Reaction (MLR), SPMCs from the different donors were mixed together 1:1 with the addition of the stimulatory molecules. Co-cultures were maintained for 5 days at 37°C for further analysis.

#### Enzyme-Linked Immunosorbent assay (ELISA)

Supernatants from PCO and SPMC co-cultures were collected 5 days after the cells were plated. Cytokine secretion levels (IFN-ɣ, TNF-α, IL-6, IL-10, IL-13, IL-1β, IL-2, IL-4, IL-8, IL-12p70) were measured for each condition in duplicates with commercially available 10-plex Human Proinflammatory Panel (MesoScale Discovery, K15049D-2) in accordance with the instructions of the manufacturers. The electrochemiluminescence readings were measured using s600 reader (MesoScale Discovery). Results are presented as mean±SEM (standard error of the mean).

#### Histology and immunofluorescence

Immediately after euthanasia by cervical dislocation, kidneys were carefully retrieved and submerged in 10% neutral buffered formalin (CellStor Pot, CellPath) for 24 hours and subsequently embedded in paraffin. 5μm serial sections were produced throughout the paraffinized kidneys. For immunostaining, slides were deparaffinized in xylene, dehydrated in graded alcohols, and rinsed with ddH2O and Tris Buffered Saline (TBS, pH 7.6). Antigen retrieval was achieved in 1mM EDTA (Sigma, E5134-500g), 10mM Tris Base (Sigma, 93352) and 0.05% Tween-20 (Sigma, P9416) buffer pH 9.0 at 96°C for 30 minutes, followed by 30 minutes cooling at room temperature. Slides were washed with TBS and blocked for 30 minutes with 10% Normal Donkey Serum (Abcam, ab138579) diluted in TBS containing 5% (w/v) IgG and protease-free bovine serum albumin (Jackson Immunoresearch, 001-000-162) in a humidified chamber. Primary antibodies diluted in the blocking buffer were incubated overnight at 4°C: human CD45 (1:100, BD Biosciences, 555480, clone [HI30]), human KRT-7 (1:200, abcam, ab68459, clone [EPR1619Y]) or human HLA-DP DQ DR (1:100, Agilent, M0775, clone [CR3/43]). Secondary antibodies (donkey anti-rabbit IgG (H+L) Alexa Fluor 555 A31572, donkey anti-mouse IgG (H+L) Alexa Fluor 647 A31571 or donkey anti-mouse IgG (H+L) Alexa Fluor 488 A21202 (all from Thermo Fisher Scientific) diluted 1:200 and DAPI-Hoechst 33342 (1:300, Invitrogen, H3570) in blocking buffer, were incubated 1 hour at room temperature. Sections were mounted in Fluorescence Mounting Medium (Agilent, S3023) under a 24x50 mm coverslip. Images were taken with an Olympus IX81 fluorescence inverted microscope. Post-acquisition analysis was performed using ImageJ v2.0 software.

#### Immunohistochemistry

Following baking at 60°C for 1 h, sections were dewaxed and rehydrated on Leica’s automated ST5020. The staining was performed on Leica’s automated Bond-III platform in conjunction with their Polymer Refine Detection System (DS9800) and a modified version of their standard template for human CD45 antibody (1:250, DAKO, M0701, clone [2B11 + PD7/26]) using Tris EDTA (Leica’s Epitope Retrieval Solution 2, AR9640) pre-treatment at 100°C for 20 minutes, followed by Protein Block (DAKO, X090930-2), and DAB Enhancer (Leica, AR9432) for 10 minutes at room temperature. Next, de-hydration and clearing were performed on Leica’s automated ST5020 and sections were mounted on Leica’s coverslipper CV5030 with mounting media DPX Mountant for Histology (Sigma Aldrich, 06522-500ML) and 22501.5 micro-coverglass coverslips (Leica Microsystems, 3800121G).

#### Opal panel staining

Sections were baked for 1 hour at 60°C. Slides were then loaded onto Leica’s automated Bond Rx platform, where they received additional baking and were dewaxed using Leica’s ‘Bake and Dewax’ preparation protocol. Following a 20 minutes ER2 pre-treatment at 100°C (ER2: Leica’s Epitope Retrieval Solution 2, AR9640; this is Tris EDTA), multiplex immunofluorescnce staining was performed sequentially, in conjunction with Akoya Biosciences Opal 6-Plex Detection Kit – for Whole Slide Imaging (reference: NEL871001KT). The sequence of staining and specific details can be found in Table S7. Finally, sections were counterstained with Spectral DAPI, removed from the Leica Bond and aqueously mounted in ProLong Diamond Antifade Mountant (Invitrogen, P36970). Stained slides were scanned with 20x magnification (resolution 0.5μm/pixel) on the PhenoImager HT (Akoya Biosciences).

#### Mass cytometry cell preparation

The preparation of the SPMC for Cytometry by Time-Of-Flight (CyTOF) was carried out according to the Maxpar Human Immune Monitoring Panel Kit (Fuidigm, 201234). Briefly, cells were washed with 10mL of Maxpar Cell Staining Buffer (Fluidigm, 201068) then centrifuged at 300g for 5 minutes. The pellet was resuspended in Maxpar Cell Staining Buffer to a final concentration of 6x10^7^ cells/mL and Human TruStain FcX (BioLegend, 422302) was added and incubated for 10 minutes at RT to block the Fc receptor. The FcR blocked cells were then transferred into a 5mL tube containing the dry antibody pellet. Mouse CD45 (Gold 156) was added to the antibody panel to identify mouse cells. A list of surface markers used can be found in manufacturer’s commercial protocol. After 30 minutes of incubation at RT, the cells were washed twice with 3mL of Maxpar Cell Staining Buffer and centrifugation at 300g for 5 minutes. The cells were fixed by incubation for 10 minutes at RT with 1.6% formaldehyde solution. The cells were centrifuged at 800g for 5 minutes and resuspended in 250nM iridium intercalator (DNA staining) and incubated overnight at 4°C. Before acquisition on the Helios mass cytometer (Fluidigm), the samples were washed and resuspended in Maxpar Cell Acquisition Solution (Fluidigm, 201240) containing 0.1X EQ Four Element Calibration beads (Fluidigm, 201078).

#### Spatial Transcriptomics

One representative mouse per group was selected, PCO and matrigel-only kidneys were sectioned (5mm) and slides sent to NanoString (Seattle, US) for analysis. The slide preparation for WTA followed standard procedure. Briefly, for WTA assay, after baking for 3 hours in 65°C, slides were processed on Leica automation platform with a protocol includes three major steps: 1) slide baking and dewax, 2) Antigen Retrieval for 20 minutes at 100°C, 3) 1μg/ml Proteinase K treatment for 20 minutes. Slides were then incubated with GeoMx WTA assay probe cocktail overnight. The following day, slides were washed and incubated with morphology markers before loading onto the GeoMx machine (Nanostring, Bruker). The conjugated antibodies used were: KRT-7 (1:200, abcam, ab215855, clone [KRT7/760]), CD3 (1:100, Origene, UM000048BF, clone [UMAB54]), CD45 (1:200, CST, 13917BF, clone [D9M8I]), in addition to nuclear staining (Syto 83, ThermoFisher Scientific, S11364).

On the GeoMx machine, slides were scanned for fluorescence and Region of Interest (ROI) were selected. Barcodes were subsequently collected followed by barcoding reading done with Illumina NGS platform.

#### DNA sequencing and sequence read Alignment

Sequencing libraries for nanorate sequencing (NanoSeq) were prepared as previously described by Abascal et al.^37^ Samples were multiplexed and sequenced on an Illumina NovaSeq 6000 platform to generate 150-base-pair (bp) paired-end reads. For each sample, DNA sequence reads were aligned to the reference human genome assembly (version GRCh37/hg19) using the BWA-MEM algorithm^65^ as implemented in BWA (v0.7.17-r1188), with options ‘-T 30 -Y -p -t 8’. The aligned reads were sorted and indexed using samtools (v1.9),^66^ and duplicate reads were marked using the bammarkduplicates tool from biobambam2 (v2.0.87; gitlab.com/german.tischler/biobambam2). Reads were additionally processed using the computational workflow described by Abascal et al.^37^ (github.com/cancerit/NanoSeq).

#### Bioinformatics software

GeoMx Spatial Transcriptomics data analysis was performed using R 4.2.2. Other analyses were performed using Python 3.7.4. The following modules were used: jupyterlab 1.1.4, loompy 3.0.6, louvain 0.6.1, matplotlib 3.3.1, numpy 1.19.1, pandas 0.25.1, pyscenic 0.10.4, python-igraph 0.7.1, python-louvain 0.13, scanpy 1.4.5, scikit-learn 0.23.2, scipy 1.5.2, scvelo 0.1.25, seaborn 0.9.0, umap-learn 0.4.0, velocyto 0.17.17. Mutation data analyses were performed using the following software: biobambam2 2.0.87, BWA-MEM 0.7.17-r1188, NanoSeq workflow 3.5.4, samtools 1.9. Analyses of mutation data were performed on R 4.0.2, using the package dndscv 0.0.1.0.

### Quantification and statistical analysis

#### Mass cytometry data analysis

Following data acquisition, FCS files were normalized based on the equilibration beads (EQ Four Element Calibration beads, 201078, Fluidigm) added to each sample, using Fluidigm CyTOF software. Data analysis was done using OMIQ software (www.omiq.ai). Debris, dead cells and QC beads were negative for the DNA1 and DNA2 channels and removed during the data clean-up. Human cells were selected as the human CD45^+^ cells (for humanized mouse samples) and a combination of dimensionality reduction and clustering analysis was performed on this population. Opt-SNE algorithm was performed for dimensionality reduction and cells were then clustered using FlowSOM. FlowSOM metaclusters were overlaid on the opt-SNE map and meta-clusters were identified by visual inspection of marker expression within each metacluster.

#### Human CD45 quantification

Whole slide scanning was performed on the Leica Aperio AT2 at a resolution of 0.5μm/pixel (*n* = 2 per group). Scanned images in the.svs file format were uploaded into HALOImage Analysis Platform version 3.6.4134 and HALO AI version 3.6.4134 (Indica Labs, Inc.). Regions for analysis were annotated manually using HALO software tools. The Multiplex IHC v3.4.9 module was used to count total cells and CD45^+^ cells. The CD45 minimum intensity for the positive threshold was set to 0.054 AU.

#### Spatial transcriptomics analysis

Cell population quantification was performed on Q3 normalized spatial transcriptomics data using SpatialDecon (ver. 1.6.0) and SafeTME^33^ or PBMC datasets (https://singlecell.broadinstitute.org/single_cell/study/SCP345/ica-blood-mononuclear-cells-2-donors-2-sites#study-summary). Differential expression analysis of HLA groups and their control was executed using DESeq2 (ver. 1.36.0) and pathway enrichment analysis was performed with fgsea (ver. 1.25.1) based on the differential expressed genes (absolute Fold Change ≥ 1, *p*-value ≤ 0.01) for either hCD45+ or hKRT-7+ compartments. The changes between the HLA groups and their control were represented by their differences in average proportion across the ROIs.

The ABMR gene panel was generated based on the NanoString Banff Human Organ transplant AMR^34^^,^^35^ and ABMR-Rat^36^ together with gene significantly highly expressed in the ABMR group of the GSE36059 dataset. The TCMR gene was generated based on the BHOT TCMR panel and genes significantly highly expressed in the TCMR group compared to non-reject in the GSE36059 dataset. Chronic and Acute TCMR gene panels were established based on the chronic TCMR group or acute TCMR vs. donor in the GSE69677 dataset. The rejection gene panel was defined by the genes associated with the Allograft Rejection terms in PathCards and KEGG (hsa05330). These gene panels were used to subset the expression value in the transcriptomics data and the changes were represented by the differences in average expression of all genes across the ROIs.

#### Mutation calling and burden analysis

For each sample, point mutations were identified using the mutation calling workflow described by Abascal et al.^37^ (github.com/cancerit/NanoSeq) with default parameters. The original human primary tissue sample was selected as the matched normal for all the samples. Mutation burdens per cell (i.e., mutations per diploid genome) were estimated using the method described by Abascal et al.^37^ (github.com/cancerit/NanoSeq). Briefly, the number of somatic mutations identified in the sample was scaled by the ratio between the number of bases sequenced to enough depth to allow base calling, and the total number of bases in the diploid human genome, while accounting for differences in trinucleotide sequence composition between the set of sequenced bases and the reference human genome. The total numbers of mutations per cell acquired during *in vitro* culture were estimated by calculating the difference between the respective mean burden per cell for each PCO sample (P0, P1, P5) and the mean burden per cell for the primary tissue sample. The numbers of coding mutations per cell acquired *in vitro* were estimated by scaling the total numbers of mutations per cell acquired *in vitro* by the ratio between the size of the diploid human genome (6.2 Gb) and the size of the diploid protein-coding human genome (69,553,910 bp), which was obtained from the CDS database (RefCDS) of the dNdScv (v0.0.1.0) R package.^67^

#### Statistical analyses

Statistical analyses were conducted using GraphPad Prism 10 and R software (versions 4.0.2 and 4.2.2). Results are presented as mean ± standard error of the mean (SEM). Specifically, ANOVA (one-way) and post hoc multiple comparisons using Tukey test were performed to assess the cytokine secretion differences of the *in vitro* co-cultures and *in vivo* hCD45+ infiltration. Paired and unpaired t-tests were used to analyse autologous response influenced by viability and cell matrix composition, respectively, in *in vitro* co-cultures. Confidence intervals (90%) were calculated by taking all hCD45+ cells (%) quantified in multiple areas per mouse per group. The number (n) of samples, replicates, p-values and animals included in each analysis is indicated in the respective Figure Legends or section in STAR Methods.

## Supplemental information

Document S1. Figures S1–S6 and Tables S1, S2, and S7

Table S3. Secretion results for 10 analytes after PCO and SPMC co-culture for 5 days, related to Figure 2

Table S4. Mass cytometry results for original donors and humanized mouse groups, related to Figure 3

Table S5. Quantification dataset of hCD45+ cells in Autologous, Partial Match, Full Mismatch, and Non-Humanized groups, related to Figure 4

Table S6. Differential expression results for hCD45+ and KRT-7+ compartment comparing autologous, partial match, and full mismatch groups, related to Figure 5

Document S2. Article plus supplemental information

## References

[1] Blau H.M., Daley G.Q. (2019). Stem Cells in the Treatment of Disease. N. Engl. J. Med..

[2] Hoang D.M., Pham P.T., Bach T.Q., Ngo A.T.L., Nguyen Q.T., Phan T.T.K., Nguyen G.H., Le P.T.T., Hoang V.T., Forsyth N.R. (2022). Stem cell-based therapy for human diseases. Signal Transduct. Target. Ther..

[3] Aly R.M. (2020). Current state of stem cell-based therapies: an overview. Stem Cell Investig..

[4] Yamanaka S. (2020). Pluripotent Stem Cell-Based Cell Therapy—Promise and Challenges. Cell Stem Cell.

[5] (2018). Method of the Year 2017: Organoids. Nat. Methods.

[6] Artegiani B., Clevers H. (2018). Use and application of 3D-organoid technology. Hum. Mol. Genet..

[7] Tang X.-Y., Wu S., Wang D., Chu C., Hong Y., Tao M., Hu H., Xu M., Guo X., Liu Y. (2022). Human organoids in basic research and clinical applications. Signal Transduct. Target. Ther..

[8] Nguyen H.T., Jacobs K., Spits C. (2018). Human pluripotent stem cells in regenerative medicine: where do we stand?. Reproduction.

[9] Mount N.M., Ward S.J., Kefalas P., Hyllner J. (2015). Cell-based therapy technology classifications and translational challenges. Philos. Trans. R. Soc. Lond. B Biol. Sci..

[10] Petrus-Reurer S., Romano M., Howlett S., Jones J.L., Lombardi G., Saeb-Parsy K. (2021). Immunological considerations and challenges for regenerative cellular therapies. Commun. Biol..

[11] Cao J., Li X., Lu X., Zhang C., Yu H., Zhao T. (2014). Cells derived from iPSC can be immunogenic - yes or no?. Protein Cell.

[12] Scheiner Z.S., Talib S., Feigal E.G. (2014). The potential for immunogenicity of autologous induced pluripotent stem cell-derived therapies. J. Biol. Chem..

[13] Liu X., Li W., Fu X., Xu Y. (2017). The Immunogenicity and Immune Tolerance of Pluripotent Stem Cell Derivatives. Front. Immunol..

[14] Chhabra A. (2017). Inherent Immunogenicity or Lack Thereof of Pluripotent Stem Cells: Implications for Cell Replacement Therapy. Front. Immunol..

[15] Pearl J.I., Kean L.S., Davis M.M., Wu J.C. (2012). Pluripotent stem cells: immune to the immune system?. Sci. Transl. Med..

[16] Zhao T., Zhang Z.-N., Rong Z., Xu Y. (2011). Immunogenicity of induced pluripotent stem cells. Nature.

[17] Zhao T., Zhang Z.-N., Westenskow P.D., Todorova D., Hu Z., Lin T., Rong Z., Kim J., He J., Wang M. (2015). Humanized Mice Reveal Differential Immunogenicity of Cells Derived from Autologous Induced Pluripotent Stem Cells. Cell Stem Cell.

[18] Guha P., Morgan J.W., Mostoslavsky G., Rodrigues N.P., Boyd A.S. (2013). Lack of Immune Response to Differentiated Cells Derived from Syngeneic Induced Pluripotent Stem Cells. Cell Stem Cell.

[19] Itakura G., Ozaki M., Nagoshi N., Kawabata S., Nishiyama Y., Sugai K., Iida T., Kashiwagi R., Ookubo T., Yastake K. (2017). Low immunogenicity of mouse induced pluripotent stem cell-derived neural stem/progenitor cells. Sci. Rep..

[20] Todorova D., Kim J., Hamzeinejad S., He J., Xu Y. (2016). Brief report: Immune microenvironment determines the immunogenicity of induced pluripotent stem cell derivatives. Stem Cell..

[21] Boyd A.S., Rodrigues N.P., Lui K.O., Fu X., Xu Y. (2012). Concise review: Immune recognition of induced pluripotent stem cells. Stem Cell..

[22] Bashor C.J., Hilton I.B., Bandukwala H., Smith D.M., Veiseh O. (2022). Engineering the next generation of cell-based therapeutics. Nat. Rev. Drug Discov..

[23] Kim J.J., Fuggle S.V., Marks S.D. (2021). Does HLA matching matter in the modern era of renal transplantation?. Pediatr. Nephrol..

[24] Zachary A.A., Leffell M.S. (2016). HLA Mismatching Strategies for Solid Organ Transplantation - A Balancing Act. Front. Immunol..

[25] Hafeez M.S., Awais S.B., Razvi M., Bangash M.H., Hsiou D.A., Malik T.H., Haq M.U., Awan A.A.Y., Rana A.A. (2023). HLA mismatch is important for 20-year graft survival in kidney transplant patients. Transpl. Immunol..

[26] Petrus-Reurer S., Lederer A.R., Baqué-Vidal L., Douagi I., Pannagel B., Khven I., Aronsson M., Bartuma H., Wagner M., Wrona A. (2022). Molecular profiling of stem cell-derived retinal pigment epithelial cell differentiation established for clinical translation. Stem Cell Rep..

[27] Veres A., Faust A.L., Bushnell H.L., Engquist E.N., Kenty J.H.-R., Harb G., Poh Y.-C., Sintov E., Gürtler M., Pagliuca F.W. (2019). Charting cellular identity during human in vitro β-cell differentiation. Nature.

[28] Friedman C.E., Nguyen Q., Lukowski S.W., Helfer A., Chiu H.S., Miklas J., Levy S., Suo S., Han J.-D.J., Osteil P. (2018). Single-Cell Transcriptomic Analysis of Cardiac Differentiation from Human PSCs Reveals HOPX-Dependent Cardiomyocyte Maturation. Cell Stem Cell.

[29] Sampaziotis F., Justin A.W., Tysoe O.C., Sawiak S., Godfrey E.M., Upponi S.S., Gieseck R.L., de Brito M.C., Berntsen N.L., Gómez-Vázquez M.J. (2017). Reconstruction of the mouse extrahepatic biliary tree using primary human extrahepatic cholangiocyte organoids. Nat. Med..

[30] Tysoe O.C., Justin A.W., Brevini T., Chen S.E., Mahbubani K.T., Frank A.K., Zedira H., Melum E., Saeb-Parsy K., Markaki A.E. (2019). Isolation and propagation of primary human cholangiocyte organoids for the generation of bioengineered biliary tissue. Nat. Protoc..

[31] Sampaziotis F., Muraro D., Tysoe O.C., Sawiak S., Beach T.E., Godfrey E.M., Upponi S.S., Brevini T., Wesley B.T., Garcia-Bernardo J. (2021). Cholangiocyte organoids can repair bile ducts after transplantation in the human liver. Science.

[32] Matas-Céspedes A., Brown L., Mahbubani K.T., Bareham B., Higgins J., Curran M., de Haan L., Lapointe J.-M., Stebbings R., Saeb-Parsy K. (2020). Use of human splenocytes in an innovative humanised mouse model for prediction of immunotherapy-induced cytokine release syndrome. Clin. Transl. Immunol..

[33] Danaher P., Kim Y., Nelson B., Griswold M., Yang Z., Piazza E., Beechem J.M. (2022). Advances in mixed cell deconvolution enable quantification of cell types in spatial transcriptomic data. Nat. Commun..

[34] Halloran P.F., Chang J., Famulski K., Hidalgo L.G., Salazar I.D.R., Merino Lopez M., Matas A., Picton M., de Freitas D., Bromberg J. (2015). Disappearance of T Cell-Mediated Rejection Despite Continued Antibody-Mediated Rejection in Late Kidney Transplant Recipients. J. Am. Soc. Nephrol..

[35] Curci C., Sallustio F., Serino G., De Palma G., Trpevski M., Fiorentino M., Rossini M., Quaglia M., Valente M., Furian L. (2016). Potential role of effector memory T cells in chronic T cell-mediated kidney graft rejection. Nephrol. Dial. Transplant..

[36] Smith R.N. (2021). In-silico performance, validation, and modeling of the Nanostring Banff Human Organ transplant gene panel using archival data from human kidney transplants. BMC Med. Genom..

[37] Abascal F., Harvey L.M.R., Mitchell E., Lawson A.R.J., Lensing S.V., Ellis P., Russell A.J.C., Alcantara R.E., Baez-Ortega A., Wang Y. (2021). Somatic mutation landscapes at single-molecule resolution. Nature.

[38] Behjati S., Huch M., van Boxtel R., Karthaus W., Wedge D.C., Tamuri A.U., Martincorena I., Petljak M., Alexandrov L.B., Gundem G. (2014). Genome sequencing of normal cells reveals developmental lineages and mutational processes. Nature.

[39] Rouhani F.J., Nik-Zainal S., Wuster A., Li Y., Conte N., Koike-Yusa H., Kumasaka N., Vallier L., Yusa K., Bradley A. (2016). Mutational History of a Human Cell Lineage from Somatic to Induced Pluripotent Stem Cells. PLoS Genet..

[40] Zou X., Owusu M., Harris R., Jackson S.P., Loizou J.I., Nik-Zainal S. (2018). Validating the concept of mutational signatures with isogenic cell models. Nat. Commun..

[41] Hu X., Gattis C., Olroyd A.G., Friera A.M., White K., Young C., Basco R., Lamba M., Wells F., Ankala R. (2023). Human hypoimmune primary pancreatic islets avoid rejection and autoimmunity and alleviate diabetes in allogeneic humanized mice. Sci. Transl. Med..

[42] Yoshihara E., O’Connor C., Gasser E., Wei Z., Oh T.G., Tseng T.W., Wang D., Cayabyab F., Dai Y., Yu R.T. (2020). Immune-evasive human islet-like organoids ameliorate diabetes. Nature.

[43] Hu X., White K., Olroyd A.G., DeJesus R., Dominguez A.A., Dowdle W.E., Friera A.M., Young C., Wells F., Chu E.Y. (2024). Hypoimmune induced pluripotent stem cells survive long term in fully immunocompetent, allogeneic rhesus macaques. Nat. Biotechnol..

[44] Petrus-Reurer S., Winblad N., Kumar P., Gorchs L., Chrobok M., Wagner A.K., Bartuma H., Lardner E., Aronsson M., Plaza Reyes Á. (2020). Generation of Retinal Pigment Epithelial Cells Derived from Human Embryonic Stem Cells Lacking Human Leukocyte Antigen Class I and II. Stem Cell Rep..

[45] Yamasaki S., Sugita S., Horiuchi M., Masuda T., Fujii S., Makabe K., Kawasaki A., Hayashi T., Kuwahara A., Kishino A. (2021). Low Immunogenicity and Immunosuppressive Properties of Human ESC- and iPSC-Derived Retinas. Stem Cell Rep..

[46] van der Torren C.R., Zaldumbide A., Duinkerken G., Brand-Schaaf S.H., Peakman M., Stangé G., Martinson L., Kroon E., Brandon E.P., Pipeleers D., Roep B.O. (2017). Immunogenicity of human embryonic stem cell-derived beta cells. Diabetologia.

[47] Pavlinek A., Matuleviciute R., Sichlinger L., Dutan Polit L., Armeniakos N., Vernon A.C., Srivastava D.P. (2022). Interferon-γ exposure of human iPSC-derived neurons alters major histocompatibility complex I and synapsin protein expression. Front. Psychiatr..

[48] Stripecke R., Münz C., Schuringa J.J., Bissig K.-D., Soper B., Meeham T., Yao L.-C., Di Santo J.P., Brehm M., Rodriguez E. (2020). Innovations, challenges, and minimal information for standardization of humanized mice. EMBO Mol. Med..

[49] Allen T.M., Brehm M.A., Bridges S., Ferguson S., Kumar P., Mirochnitchenko O., Palucka K., Pelanda R., Sanders-Beer B., Shultz L.D. (2019). Humanized immune system mouse models: progress, challenges and opportunities. Nat. Immunol..

[50] Shultz L.D., Brehm M.A., Garcia-Martinez J.V., Greiner D.L. (2012). Humanized mice for immune system investigation: progress, promise and challenges. Nat. Rev. Immunol..

[51] Lan X., Zhang M.-M., Pu C.-L., Guo C.-B., Kang Q., Li Y.-C., Dai X.-K., Deng Y.-H., Xiong Q., Ren Z.-M. (2010). Impact of human leukocyte antigen mismatching on outcomes of liver transplantation: a meta-analysis. World J. Gastroenterol..

[52] Charmetant X., Pettigrew G.J., Thaunat O. (2024). Allorecognition unveiled: Integrating recent breakthroughs into the current paradigm. Transpl. Int..

[53] Siu J.H.Y., Surendrakumar V., Richards J.A., Pettigrew G.J. (2018). T cell allorecognition pathways in solid organ transplantation. Front. Immunol..

[54] Cassano A., Chong A.S., Alegre M.-L. (2023). Tregs in transplantation tolerance: role and therapeutic potential. Front. Transplant..

[55] Chan Y.L.T., Zuo J., Inman C., Croft W., Begum J., Croudace J., Kinsella F., Maggs L., Nagra S., Nunnick J. (2018). NK cells produce high levels of IL-10 early after allogeneic stem cell transplantation and suppress development of acute GVHD. Eur. J. Immunol..

[56] Bogomiakova M.E., Sekretova E.K., Anufrieva K.S., Khabarova P.O., Kazakova A.N., Bobrovsky P.A., Grigoryeva T.V., Eremeev A.V., Lebedeva O.S., Bogomazova A.N., Lagarkova M.A. (2023). iPSC-derived cells lack immune tolerance to autologous NK-cells due to imbalance in ligands for activating and inhibitory NK-cell receptors. Stem Cell Res. Ther..

[57] Rossbach B., Hariharan K., Mah N., Oh S.-J., Volk H.-D., Reinke P., Kurtz A. (2022). Human iPSC-Derived Renal Cells Change Their Immunogenic Properties during Maturation: Implications for Regenerative Therapies. Cells.

[58] Benabdallah B., Désaulniers-Langevin C., Colas C., Li Y., Rousseau G., Guimond J.V., Haddad E., Beauséjour C. (2019). Natural Killer Cells Prevent the Formation of Teratomas Derived From Human Induced Pluripotent Stem Cells. Front. Immunol..

[59] Kuijk E., Jager M., van der Roest B., Locati M.D., Van Hoeck A., Korzelius J., Janssen R., Besselink N., Boymans S., van Boxtel R., Cuppen E. (2020). The mutational impact of culturing human pluripotent and adult stem cells. Nat. Commun..

[60] Merkle F.T., Ghosh S., Kamitaki N., Mitchell J., Avior Y., Mello C., Kashin S., Mekhoubad S., Ilic D., Charlton M. (2017). Human pluripotent stem cells recurrently acquire and expand dominant negative P53 mutations. Nature.

[61] Zorn E., See S.B. (2022). Antibody Responses to Minor Histocompatibility Antigens After Solid Organ Transplantation. Transplantation.

[62] Wojciechowski D., Wiseman A. (2021). Long-Term Immunosuppression Management: Opportunities and Uncertainties. Clin. J. Am. Soc. Nephrol..

[63] Abou-Khalil R., Colnot C. (2014). Renal capsule transplantations to assay skeletal angiogenesis. Methods Mol. Biol..

[64] Park H.-C., Yasuda K., Kuo M.-C., Ni J., Ratliff B., Chander P., Goligorsky M.S. (2010). Renal capsule as a stem cell niche. Am. J. Physiol. Renal Physiol..

[65] Li, H. (2014). Aligning sequence reads, clone sequences and assembly con∗gs with BWA-MEM. 10.6084/M9.FIGSHARE.963153.V1.

[66] Li H., Handsaker B., Wysoker A., Fennell T., Ruan J., Homer N., Marth G., Abecasis G., Durbin R., 1000 Genome Project Data Processing Subgroup (2009). The Sequence Alignment/Map format and SAMtools. Bioinformatics.

[67] Martincorena I., Raine K.M., Gerstung M., Dawson K.J., Haase K., Van Loo P., Davies H., Stratton M.R., Campbell P.J. (2017). Universal patterns of selection in cancer and somatic tissues. Cell.

