# Supplementary material for: Immunogenicity of autologous and allogeneic human primary cholangiocyte organoid cellular therapies
Source: Cell Rep Med. 2025 Jul 7;6(7):102205. doi: 10.1016/j.xcrm.2025.102205 (PMC12281383; doi:10.1016/j.xcrm.2025.102205)
Supplement: Document S1. Figures S1–S6 and Tables S1, S2, and S7 [file mmc1.pdf]

Cell Reports Medicine, Volume *6*

Supplemental information

Immunogenicity of autologous and allogeneic human primary cholangiocyte organoid cellular therapies

Sandra Petrus-Reurer, Winnie Lei, Olivia Tysoe, Maelle Mairesse, Adrian Baez-Ortega, Julia Jones, Thomas Tan, Sylvia Rehakova, Krishnaa T. Mahbubani, Cara Brodie, Namshik Han, Inigo Martincorena, Catherine Betts, Ludovic Vallier, and Kourosh Saeb-Parsy

SUPPLEMENTAL INFORMATION

Immunogenicity of autologous and allogeneic human primary cholangiocyte organoid cellular therapies

Sandra Petrus-Reurer[1]*, Winnie Lei[1,2], Olivia Tysoe[1], Maelle Mairesse[3], Adrian Baez-Ortega[4], Julia Jones[5], Thomas Tan[1], Sylvia Rehakova[1], Krishnaa T. Mahbubani[1], Cara Brodie[5], Namshik Han[2,6,7], Inigo Martincorena[4], Catherine Betts[3], Ludovic Vallier[8,9], Kourosh Saeb-Parsy[1,10]*

[1]Department of Surgery, University of Cambridge and NIHR Cambridge Biomedical Research Centre, Cambridge, CB2 0QQ, United Kingdom
[2]Milner Therapeutics Institute, University of Cambridge, Cambridge, *CB2 0AW*, United Kingdom
[3]Clinical Pharmacology and Safety Sciences, AstraZeneca R&D, Cambridge, CB4 0WG, United Kingdom
[4]Wellcome Sanger Institute, Wellcome Genome Campus, Hinxton, CB10 1SA, United Kingdom
[5]Cancer Research UK Cambridge Institute, Cambridge, *CB2 0AW*, United Kingdom
[6]Cambridge Centre for AI in Medicine, University of Cambridge, Cambridge, *CB2 0XY*, United Kingdom
[7]Wellcome-MRC Cambridge Stem Cell Institute, University of Cambridge, Cambridge, *CB2 0AW*, United Kingdom
[8]Berlin Institute of Health, Center for Regenerative Therapies, Berlin, 13353, Germany
[9]Max-Planck-Institute for Molecular Genetics, Berlin, 14195, Germany
[10]Lead contact
*Co-corresponding authors: **sp2016@cam.ac.uk** and **ks10014@cam.ac.uk**

SUPPLEMENTAL FIGURES

Figures S1-S6

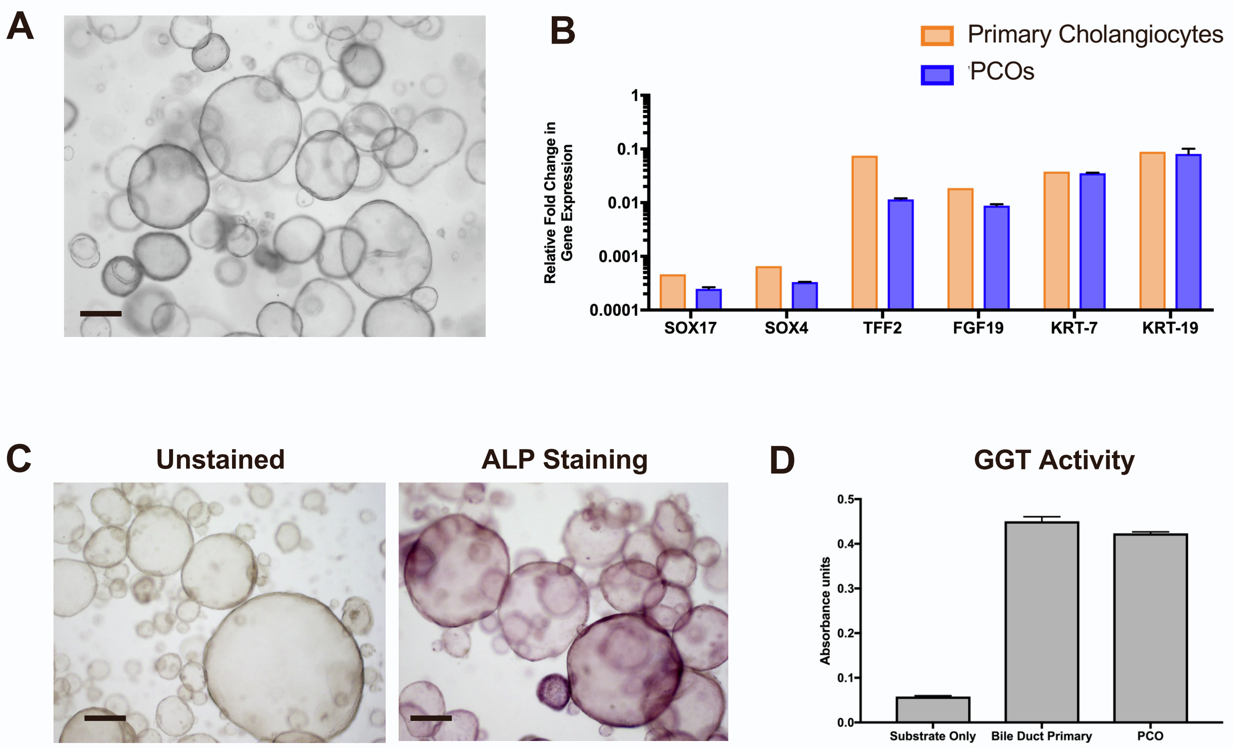

Figure S1. Characterization of cultured human primary-derived PCOs, Related to Figure 1.
(**A**) Bright field image showing cultured PCOs after passaging. (**B**) Bar graph showing RT-qPCR of cholangiocyte marker genes (*SOX17, SOX4, TFF2, FGF19, KRT-7, KRT-19*) in PCOs compared to primary bile duct tissue. Error bars represent mean±SEM from three technical replicates. (**C**) Bright field picture showing alkaline phosphatase (ALP) staining in cultured PCOs. Scale-bars: 200 μm. (**D**) Graph showing gamma-glutamyl transferase (GGT) activity of cultured PCOs compared to primary bile duct tissue and substrate only (negative control). Error bars represent mean±SEM from three technical replicates.

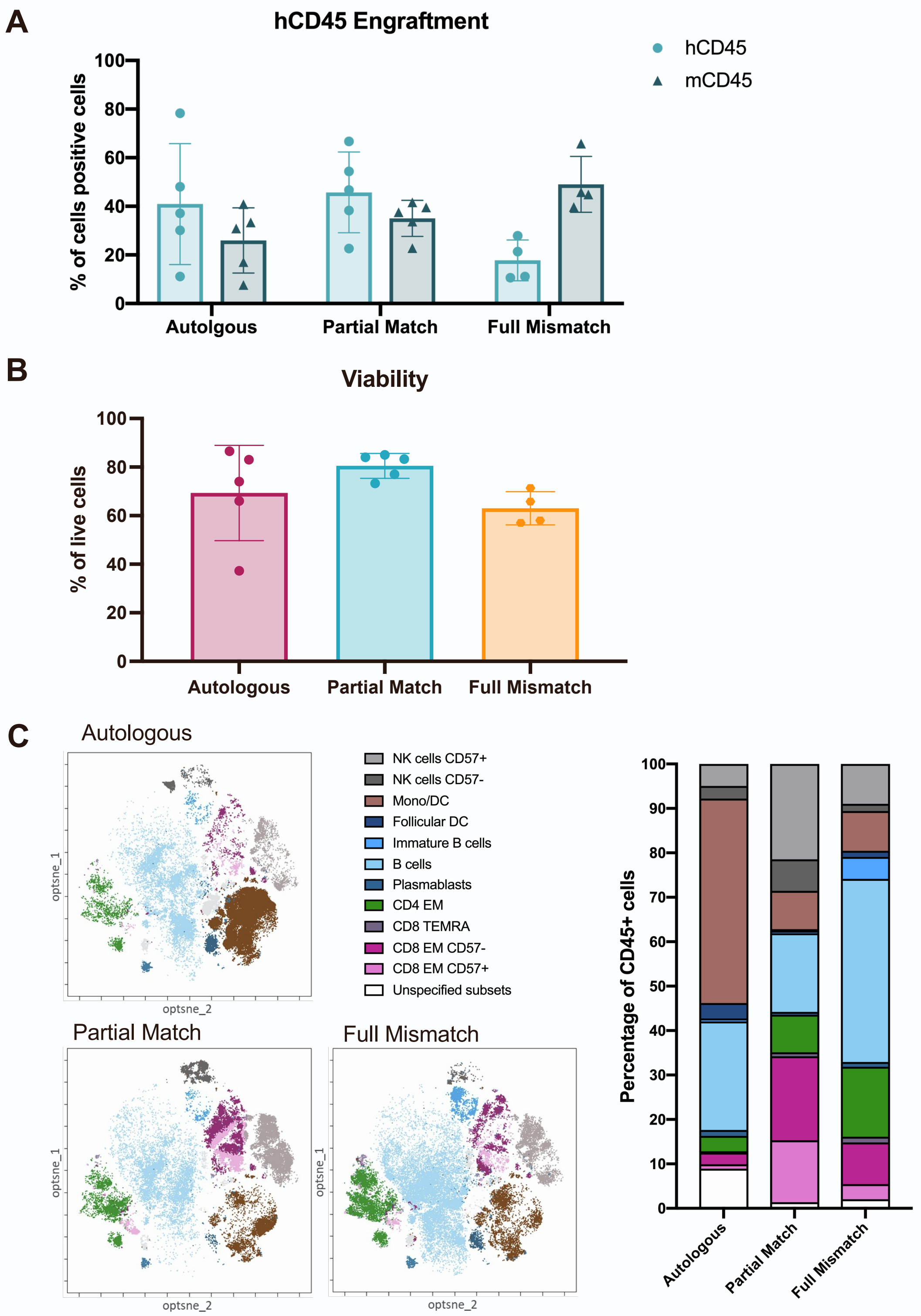

Figure S2. SPMC donor engraftment in NSG mice, Related to Figure 3.
(**A**) Bar chart showing percentage of positive cells for human and mouse CD45 cell surface marker in spleen at endpoint for Autologous, Partial Match and Full Mismatch groups (n=4-5 per group). Error bars represent mean ±SEM from 4-5 mice analyzed per group. (**B**) Bar chart showing percentage of live cells after spleen processing at endpoint for Autologous, Partial Match and Full Mismatch groups. Error bars represent mean ±SEM from 4-5 mice analyzed per group. (**C**) OptSNE visualisation and FlowSOM clustering show the immune profile of original human donor SPMCs (before engraftment) with the distribution of immune cell subpopulations in different colours. Mice with the same engraftment have been overlaid.

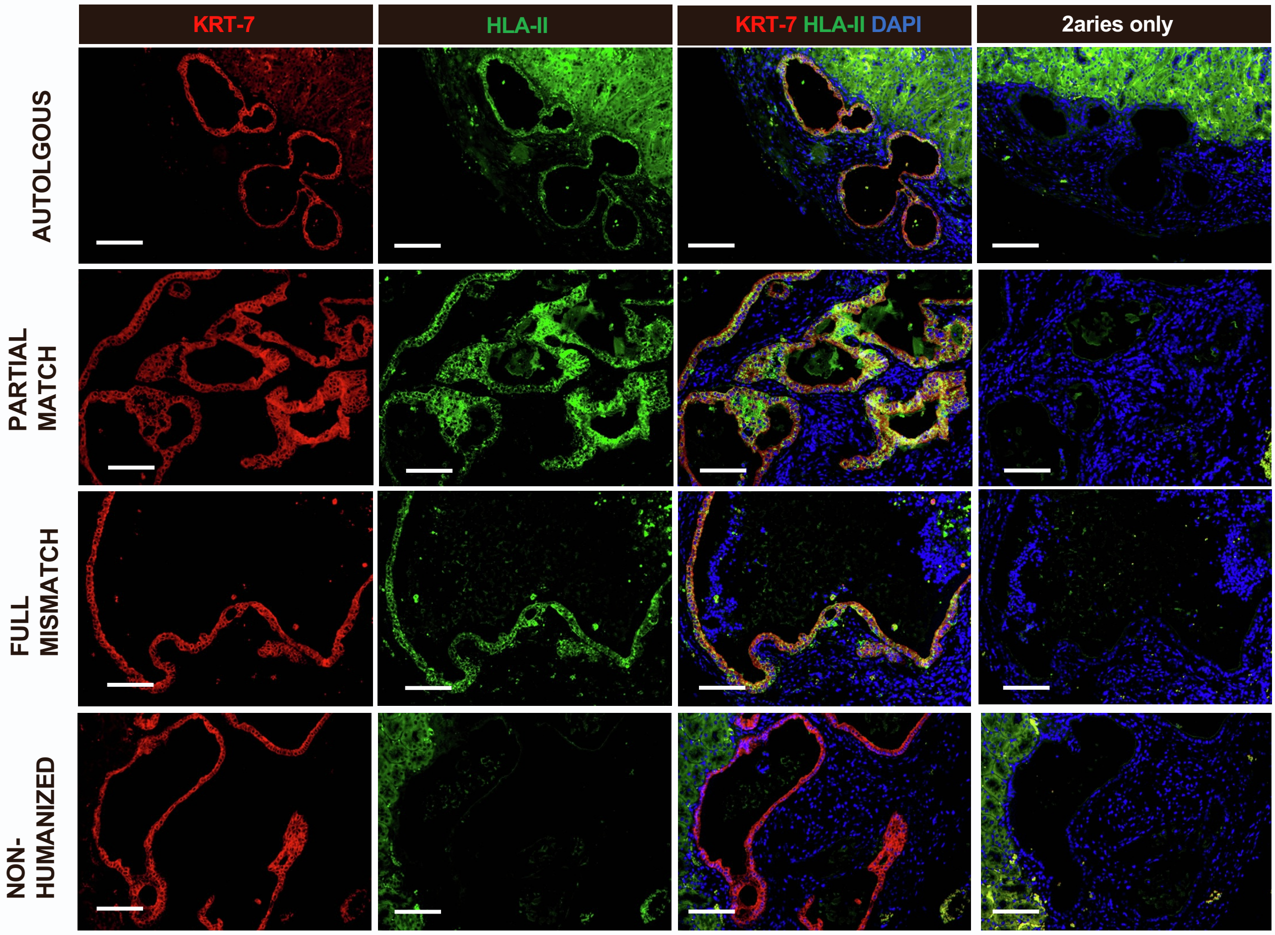

Figure S3. PCO engraftment in humanized mice, Related to Figure 4.
Immunofluorescence images of injected PCOs (passage 10) under the kidney capsule showing expression of human KRT-7 and human HLA-II markers in Autologous, Partial Match, Full Mismatch and Non-Humanized groups. Secondary antibody-only images are shown as negative control. Scale-bars: 100 µm.

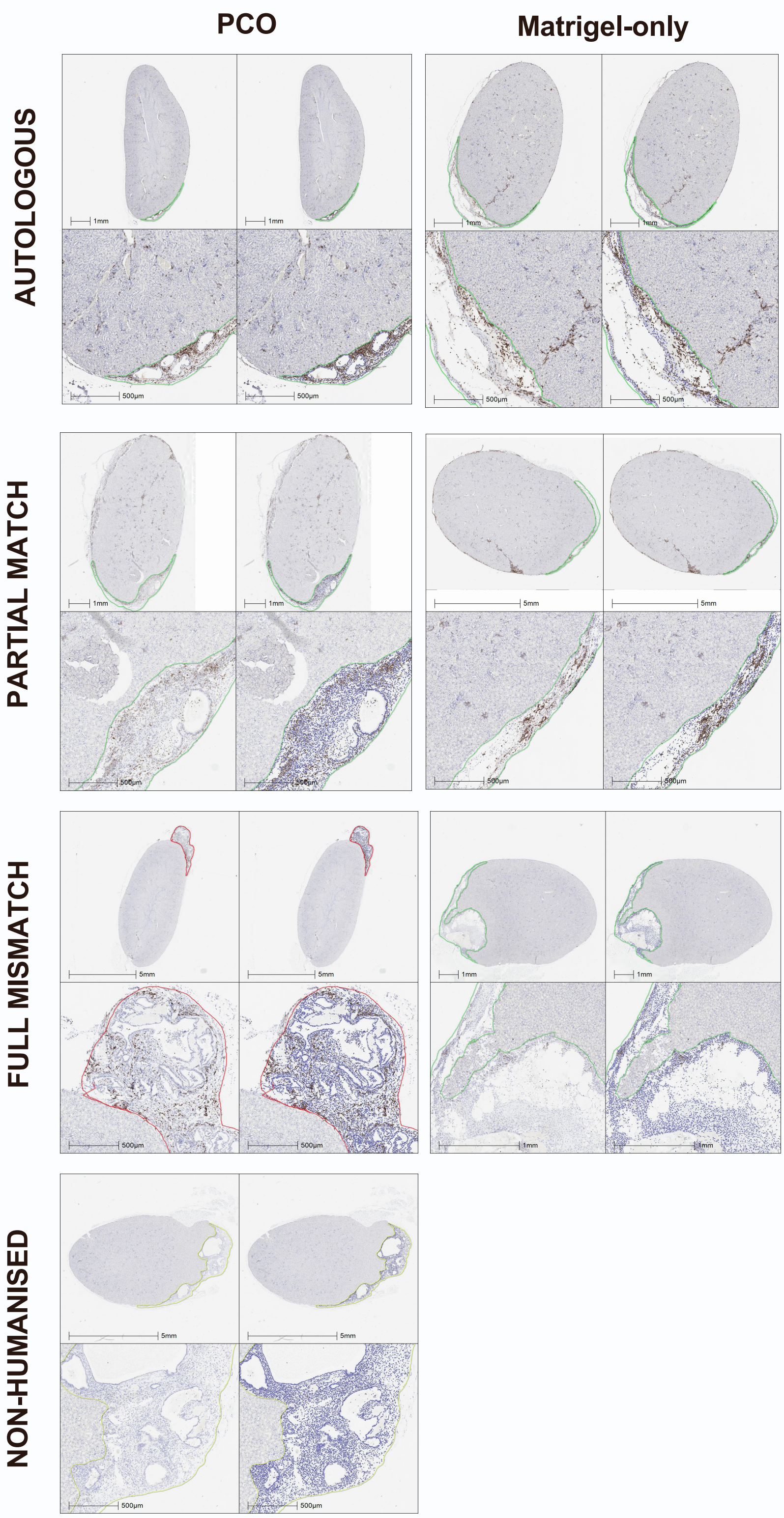

Figure S4. Segmentation of hCD45+ infiltration into PCO graft sites in humanized mice, Related to Figure 4.

Low (upper rows) and high (lower rows) magnification scans and respective custom-made segmentation showing positive human CD45 staining (brown) and hematoxylin staining (nuclei) in representative kidneys that received PCOs (left panel) or Matrigel-only (right panels) for Autologous, Partial Match, Full Mismatch and Non-Humanized groups. Marked areas in green, red and yellow are the ones used for further quantification. Scale-bars: 500µm, 1mm and 5mm.

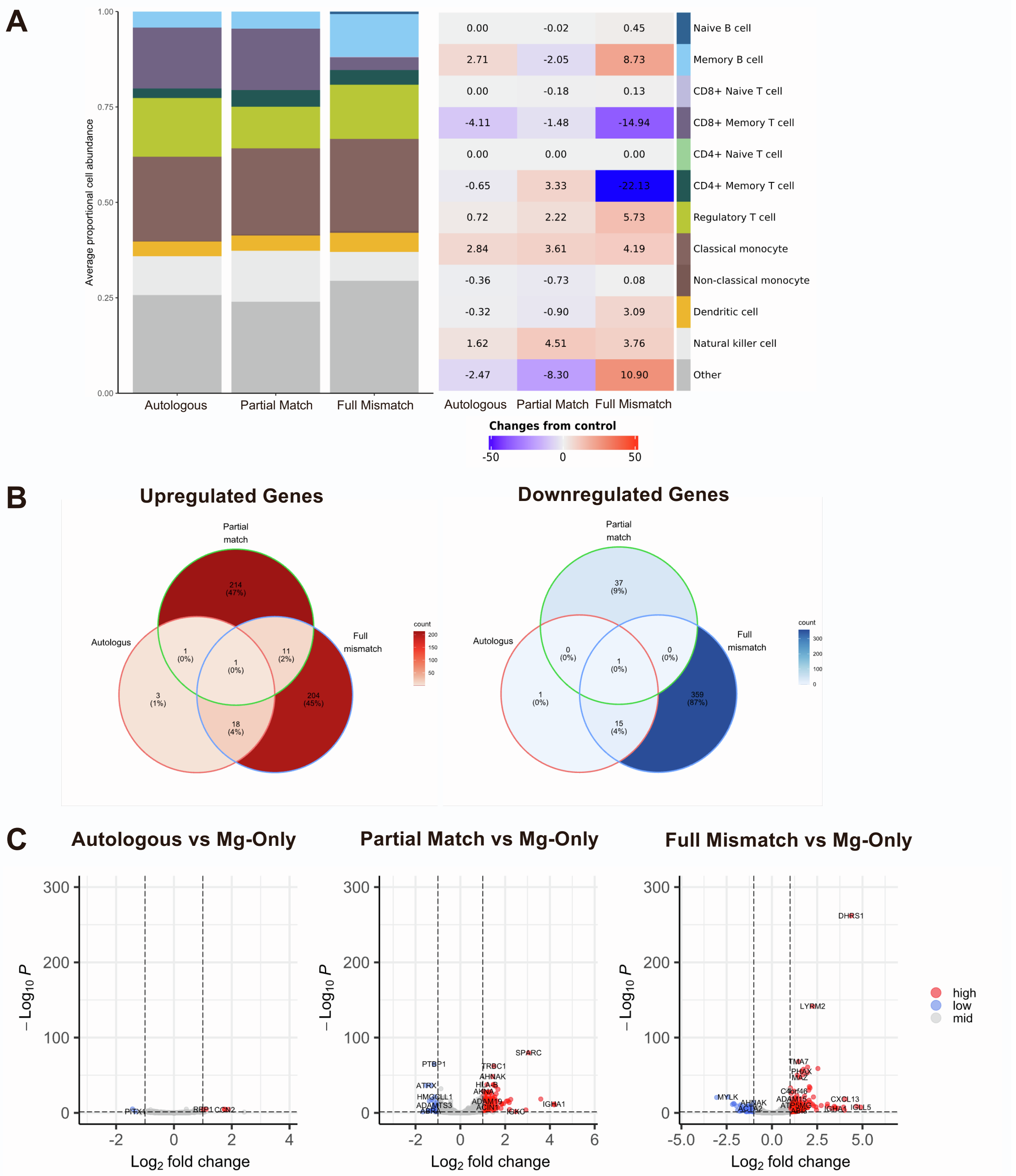

	Autologous	Partial Match	Full Mismatch
Naive B cell	0.00	-0.02	0.45
Memory B cell	2.71	-2.05	8.73
CD8+ Naive T cell	0.00	-0.18	0.13
CD8+ Memory T cell	-4.11	-1.48	-14.94
CD4+ Naive T cell	0.00	0.00	0.00
CD4+ Memory T cell	-0.65	3.33	-22.13
Regulatory T cell	0.72	2.22	5.73
Classical monocyte	2.84	3.61	4.19
Non-classical monocyte	-0.36	-0.73	0.08
Dendritic cell	-0.32	-0.90	3.09
Natural killer cell	1.62	4.51	3.76
Other	-2.47	-8.30	10.90

Figure S5. Spatial transcriptomic analysis of immune infiltration (CD45 cells) into PCO graft sites in humanized mice, Related to Figure 5.

(**A**) Histogram showing cell abundance of hCD45+ cells in Autologous, Partial Match and Full Mismatch groups (left), and their respective fold change compared to Matrigel-only controls (right). Matrix was extracted from the Single Cell Portal (study no. SCP345[1]). (**B**) Venn diagram showing upregulated and down regulated genes for Autologous, Partial Match and Full Mismatch groups. (**C**) Volcano plots showing low, mid and high differentially expressed genes in the assessed groups for hCD45+ cells compared to their respective Matrigel-only (Mg-only) control.

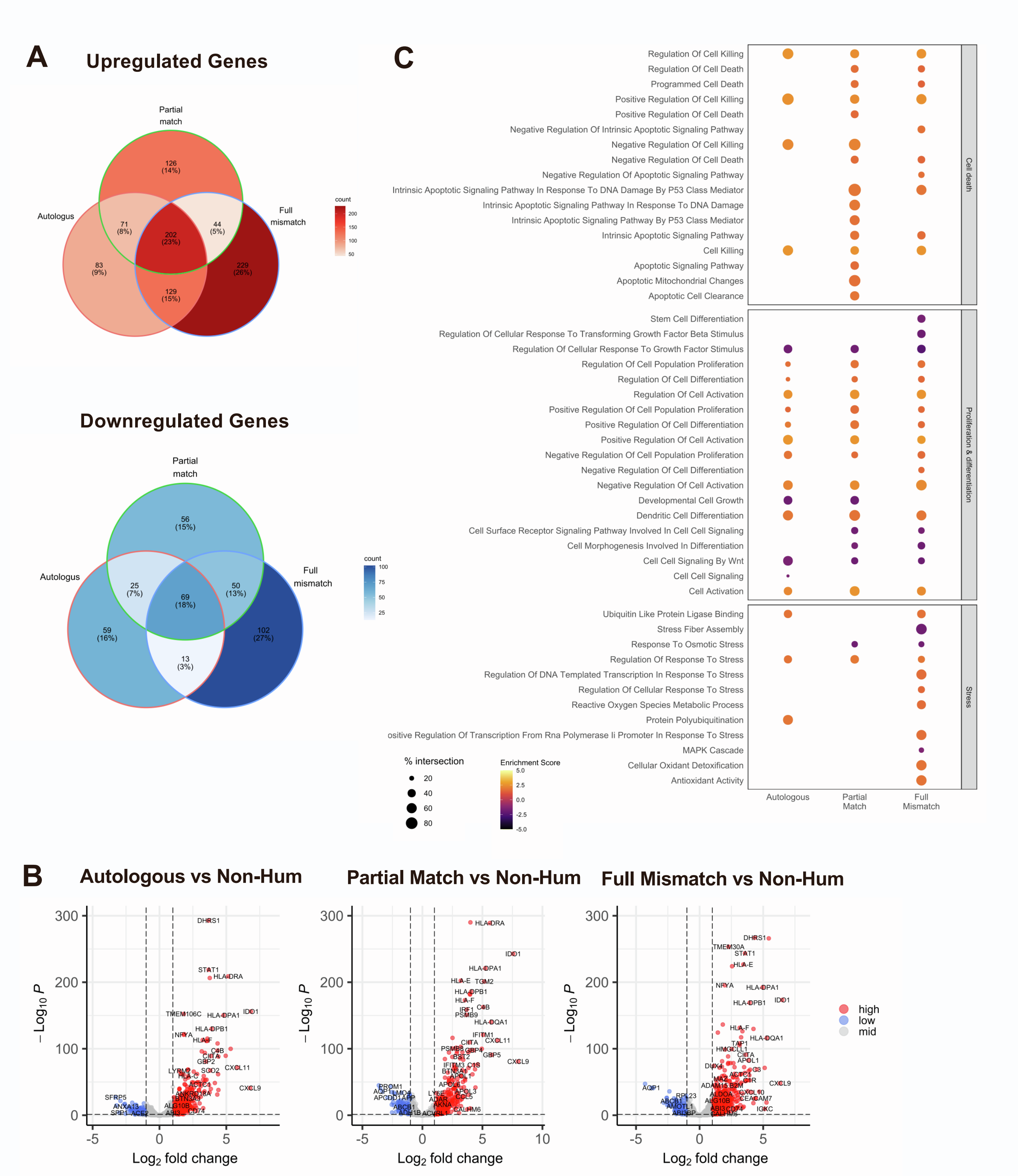

Figure S6. Spatial transcriptomics of PCO cells transplanted into humanized mice, Related to Figure 5. (**A**) Venn diagram showing upregulated and down regulated genes in KRT-7+ cells for Autologous, Partial Match and Full Mismatch groups. (**B**) Volcano plots showing low, mid and high differentially expressed genes in KRT-7+ cells compared to Non-Humanized (Non-Hum) control. (**C**) Dot plot showing enrichment analysis of Autologous, Partial Match and Full Mismatch groups for cell death, proliferation and differentiation and stress pathways in cholangiocyte cells.

SUPPLEMENTAL TABLES
Tables S1-S2, S7

Table S1. Donor demographics, Related to Figure 2 and Figure 3.

**Donor**	**Age**	**Gender**	**Ethnicity**	**BMI**	**Blood Group**	**Cause of Death**	**Donor Type**
Autologous (299B)	46 years	Male	White	30.76	O+	Hypoxic brain damage	Donation after Brainstem Death (DBD)
Partial Match (354B)	46 years	Female	White	26.71	O+	Intracraneal haemorrhage	Donation after Brainstem Death (DBD)
Full Mismatch (283)	73 years	Male	White	24.47	AB+	Intracraneal haemorrhage	Donation after Brainstem Death (DBD)

Table S2. HLA-I and HLA-II haplotypes of PCOs and SPMC donors (Autologous, Partial Match and Full Mismatch) used for humanization, Related to Figure 2 and Figure 3.

	**PCO**	**Autologous SPMC**	**Partial Match SPMC**	**Full Mismatch SPMC**
**HLA-A**	01:01	01:01	01:01	03:01
**HLA-A**	02:01	02:01	02:01	32:01
**HLA-B**	08:01	08:01	08:01	07:02
**HLA-B**	40:02	40:02	40:01	18:01
**HLA-C**	02:02	02:02	03:04	07:01
**HLA-C**	07:01	07:01	07:01	07:02
**HLA-DRB1**	03:01	03:01	03:01	09:01
**HLA-DRB1**	13:01	13:01	04:01	15:01
**HLA-DRB3/4/5**	3*01:01	3*01:01	3*01:01	4*01:03
**HLA-DRB3/4/5**	3*02:02	3*02:02	4*01:03	5*01:01
**HLA-DQA1**	01:03	01:03	03:01	01:02
**HLA-DQA1**	05:01	05:01	05:01	03:02
**HLA-DQB1**	02:01	02:01	02:01	03:03
**HLA-DQB1**	06:03	06:03	03:02	06:02
**HLA-DPA1**	01:04	01:04	01:03	01:03
**HLA-DPA1**	02:01	02:01	01:03	01:03
**HLA-DPB1**	10:01	10:01	04:01	02:01
**HLA-DPB1**	15:01	15:01	16:01	04:01

Table S7. OPAL panel staining details, Related to Star Methods.

Round	Target	Catalogue No.	Dilution/Conc.	Opal Pairing	Opal Dilution	Stripping
1	CD20	Novocastra, NCL-L-CD20-L26	0.95µg/mL	620	1:150	ER2 at 100°C for 30 min
2	CD3	ThermoScientific, RM-9107-S1	1:200	690	1:150	ER2 at 100°C for 20 min
3	FoxP3	Abcam, ab20034	19.1µg/mL	TSA-DIG, 780	1:150	N/A

REFERENCES

1. Leyla Tarhan, Jon Bistline, Jean Chang, Bryan Galloway, Emily Hanna, Eric Weitz Single Cell Portal: an interactive home for single-cell genomics data. BioRxiv. https://doi.org/10.1101/2023.07.13.548886.
